# Stochastic energy management of a microgrid incorporating two-point estimation method, mobile storage, and fuzzy multi-objective enhanced grey wolf optimizer

**DOI:** 10.1038/s41598-024-51166-9

**Published:** 2024-01-18

**Authors:** Serajuddin Habibi, Reza Effatnejad, Mahdi Hedayati, Payman Hajihosseini

**Affiliations:** Department of Electrical Engineering, Karaj Branch, Islamic Azad University, Karaj, Iran

**Keywords:** Renewable energy, Solar energy, Wind energy

## Abstract

In this study, the stochastic energy management, and scheduling of a renewable microgrid involving energy sources and dynamic storage is performed considering energy resource and demand uncertainties and demand response (DR) using the two-point estimation method (2 m + 1 PEM). The three-dimensional objective function is defined as maximizing the renewable hosting capacity and minimizing the operation cost, and emission cost minimization. The decision variables include installation location and size of the renewable resources and mobile energy storage system (MESS), determined using a multi-objective enhanced grey wolf optimizer (MOEGWO) improved based on the logistic chaotic mapping integrated with fuzzy decision-making approach. The simulations are implemented for several cases of employing MESS, DR, and uncertainties to investigate the proposed approach's efficacy. The MOEGWO performance is confirmed to solve the ZDT and CEC'09 functions according to some well-known algorithms. Then, the performance of the MOEGWO is evaluated on the stochastic energy management and scheduling of the renewable microgrid. The results indicate that considering the dynamic MESS causes reducing the operation and emission costs by 23.34% and 34.78%, respectively, and increasing the renewable hosting capacity by 7.62% in contrast to using the static MESS. Also, the stochastic problem-solving considering uncertainties showed that operation and emission costs are raised, the renewable hosting capacity is decreased, and the uncertainty impact is reduced in the condition of DR application. So, the results validated the proposed methodology's effectiveness for minimizing the operation and emission costs and maximizing the renewable hosting capacity. Moreover, the superior capability of the MOEGWO is confirmed in comparison with the multi-objective particle swarm optimization to obtain lower operation and emission costs and higher renewable hosting capacity.

## Introduction

### Motivation and research background

Future distribution systems will likely contain a greater proportion of renewable photovoltaic (PV) and wind turbine (WT) energy sources (ERs) as a result of growing environmental concerns and efforts to minimize energy costs. These resources offer numerous benefits to distribution networks. One of the most significant benefits of distributed generation (DG) based on ERs in distribution networks is that it enables the construction of microgrids^1,2^. A microgrid typically consists of multiple ERs, such as WT, PV, hydro, microturbine (MT), fuel cell (FC), biomass, natural gas generator, load, and energy storages, which can be operated independently or connected to the network. Globally, there is a wave of change toward adopting renewable energy sources, which are frequently incorporated into the power system as the DGs^3,4^. Some of these disadvantages have been discussed within the context of energy management if the resources of DGs, particularly those that depend on renewable energies, aren't being applied effectively, resulting in network operation problems such as significant resistance losses and voltage drops in transmission lines^5,6^. On the other hand, the increased presence of these resources introduces technical constraints, such as bus voltage and the amount of electricity passing through the distribution network's feeders. In addition, the hosting capacity of the distribution network is defined as the total maximum hosting capacity of ERs that can be implemented in the distribution network without exceeding the operational constraints of the distribution network^7–9^. Uncertainty in the output power of renewable resources, such as wind and photovoltaic (PV) energy, in distribution microgrids is one of the primary challenges the operator encounters in managing these sources, which impacts the network's capacity to host them. This indicates that the actual production capacity of these resources is distinct from its predicted value. Due to the uncertainty in the production energy of these resources, the network operators will need help creating a balance between power production and consumption. In addition to the uncertainty surrounding ERs, the consumption burden is also uncertain. In this manner, the sources are uncertain, and their correlation has a significant effect. Therefore, the primary challenge of grid-connected microgrids is energy management that considers the operational costs and emission costs of environmental pollutants in the presence of uncertainty and the application of optimization and uncertainty modeling techniques. In the literature^10,11^, deterministic and stochastic approaches are used to solve microgrids' scheduling and energy management. In deterministic microgrid energy management, it is assumed that the output power of renewable energy sources, the demand power, and market prices are identical to their predicted values. Several stochastic energy management input variables are uncertain. Due to the stochastic nature of wind speed and solar radiation, it is exceedingly difficult to precisely predict WT and PV output power. In addition, the predicted values of load demand and market price will not be exact due to unanticipated disturbances, forecaster errors, and fluctuations in demand and price^12^.

### Literature review and research gap

Due to the challenge of coordinating among diverse generation units, energy storage devices, and load management equipment, resolving the energy management problem of microgrids is an especially challenging undertaking. In^13^, a multi-layer ant colony optimization (ACO) is suggested to address microgrid energy management in order to establish energy scheduling in order to minimize total production costs. A multi-period artificial bee colony (ABC) method is presented in^14^ to address economic load flow taking generation, storage, and responsive load into consideration. In^15^, an efficient algorithm called particle swarm optimization (PSO) is presented to solve the microgrid energy management problem considering different energy storage units and distributed generation sources. In^16^, the energy management of multiple microgrids is implemented with the help of a common line with a common connection point to the network. In^17^, real-time energy management is developed to solve the optimal scheduling of the battery charge and discharge pattern in a microgrid by minimizing the operation and charge/discharge cost of the battery using the PSO. In^18^, the energy management of a microgrid is implemented to minimize the operating cost by considering the battery degradation using the PSO. In^19^, the energy management of a proposed integrated microgrid with WT and PV sources, diesel generator, energy storage, and CHP sources to meet electrical and thermal demands to minimize operating costs via a reinforcement learning (RL). In^20^, multi-objective optimization and energy management of a microgrid is presented to reduce energy exchange with the main grid based on the independence performance factor and also minimize power loss, pollution, and voltage drop considering DR using an epsilon-greedy algorithm (EGA). In^21^, a stochastic decision-making method based on a compromised program (CP) is presented for the energy management of a multiple microgrids with the aim of minimizing the investment costs of installing power cables and operating costs. In^22^, the optimal operation of a microgrid connected to the distribution system is developed, and the best investment and operation strategy of the CHP system, boiler, PV power generation, and battery are determined by the optimization method. In^23^, stochastic scheduling of a microgrid is implemented considering the power uncertainty of PV and WT sources using Monte Carlo simulation (MCS) based on the hybrid Jaya algorithm and interior point method (Jaya-IPM). Multi-objective microgrids operation integrated with distributed generation and combined heat and power (CHP) is devised in^24^ to minimize cost, energy loss, and voltage deviation via Mont Carlo simulation and point estimate technique with teaching–learning-based optimization and firefly algorithm (TLO-FA). In^25^, an optimal microgrid energy management method is developed to meet CHP demand by hydrogen stations, EVs, and fuel cells to minimize the operating cost incorporating the alternating direction method of multipliers (ADMM). In^26^, a microgrid scheduling model with multiple energy sources is proposed to supply the electricity, gas, and heat needs of subscribers using renewable sources and multi-energy conversion methods. In^27^, an energy microgrid operation integrated with energy resources and also electric vehicles is implemented to minimize the operation cost and also the voltage deviation considering uncertainty of the resources using GAMS software. In^28^, a stochastic scheduling method for microgrid participation in the energy market is developed by determining the scheduling of energy resources considering DR for minimizing the operating cost of electric and thermal loads using water wave optimization (WWO). In^29^, scheduling the energy management of a microgrid is performed considering uncertainty and DR to minimize the cost of operation and emission via a quantum PSO (QPSO). In^30^, the dynamic scheduling of an energy microgrid is presented based on the colored Petri net (CPN) integrated with the QPSO. In^31^, the stochastic scheduling of a microgrid based on renewable energy sources and battery energy storage is developed using the barnacles mating optimizer (BMO). In^32^, the multi-objective and stochastic scheduling of a microgrid including storage, energy sources, and DR based on clustering and scheduling layers is presented to minimize the operation cost and pollution emission using the column and constraint generation (C&CG) algorithm. Mathematical programming and meta-heuristic methods are applied to solve the microgrid scheduling and energy management in previous studies. Mathematical programming methods are capable to guarantee reaching the optimal point, while meta-heuristic methods do not guarantee this. Heuristic techniques, on the other hand, can tackle large and complex optimization problems, whereas mathematical programming techniques might not work as the complexity of the optimization problem expands. Mathematical methods are based on the derivative, in other words, the Lagrange function is calculated in them, and then the Cuhn-Tucker constraint is derived for it and the problem is solved. Differentiability is also obtained when the problem is convex, but the problem of load distribution in electrical networks such as microgrid is non-convex. Therefore, there is the problem of derivability, which of course has solutions, but they are complicated^33^. Moreover, problem solving is based on complex mathematical algorithms. But solving these problems is easily possible using meta-heuristic algorithms. For this reason, meta-heuristic algorithms have been used in this study to prevent the complexity of the problem and to prevent the increase of computational cost.

According to the literature review summarized in Table 1, the research gaps are presented as follows:

- The literature review has shown that most studies have not considered modeling uncertainties in microgrid scheduling. Due to the uncertainty of the production of renewable energy sources and also the load demand, the amount of load may be higher than the predicted value and the production may be lower than the predicted value in a deterministic scenario. Therefore, the output results are not reliable because the reserve level considered may not respond to the fluctuations of renewable resources. Moreover, in this situation, microgrid planning should be performed based on the stochastic model, taking into account the uncertainties of resource and load generation, which is less addressed in the previous researches.
- In the studies that are implemented based on the stochastic model, they used the conventional method of Monte Carlo simulation (MCS) to model the uncertainties. The MCS is a method that requires the probability distribution function (PDF) of parameters with uncertainty, while its computational cost is high and its output is highly dependent on the definition of input scenarios. The Monte Carlo method's primary drawback is the large number of simulations required to obtain convergence. Devoid of complete knowledge of the probability functions of random variables, there is a requirement for uncertainty modeling techniques that involve less computational burden and can circumvent the challenges resulting from this lack of knowledge.
- The microgrid operators try to overcome existing uncertainties and increase the hosting capacity from the DGs especially renewable energy resources contribution to have a certain amount of storage in the system. Although these problems were overcome by buying more energy from the upstream network or increasing the number of resources, it caused problems such as increasing the amount of pollution. The evaluation of the literature review has shown that the use of mobile storage for this purpose has not been well conducted. In general, mobile energy storage system (MESS) is a type of storage that is installed on a vehicle and can move in the distribution network. The effect of simultaneously using these resources with demand-side load management has not been well evaluated in previous studies.

**Table 1:** Summarize of the literature.

Ref	Determinstic/stochastic	Demand response	Dynamic (mobile) storage	Hosting capacity	Fuzzy decision making	Solver
^13^	Determinstic	Yes	No	No	No	ACO
^14^	Stochastic/Markov model	Yes	No	No	No	ABC
^15^	Determinstic	No	No	No	No	PSO
^16^	Determinstic	No	No	No	No	MILP
^17^	Determinstic	No	No	No	No	PSO
^18^	Determinstic	No	No	No	No	PSO
^19^	Determinstic	No	No	No	No	RL
^20^	Stochastic	Yes	No	No	No	EGA
^21^	Stochastic	Yes	No	No	Yes	CP
^22^	Stochastic	No	No	No	No	MILP
^23^	Stochastic	Yes	No	No	No	Jaya-IPM
^24^	Stochastic	Yes	No	No	Yes	TLO-FA
^25^	Stochastic	Yes	No	No	No	ADMM
^26^	Stochastic	Yes	No	No	No	MINLP
^27^	Stochastic	Yes	No	No	Yes	MINLP
^28^	Stochastic	Yes	No	No	No	WWO
^29^	Stochastic	Yes	No	No	No	QPSO
^30^	Determinstic	No	No	No	No	CPN-QPSO
^31^	Stochastic	No	No	No	No	BMO
^32^	Stochastic	No	No	No	Yes	C&CG
Proposed model	Stochastic	Yes	Yes	Yes	Yes	MOEGWO

### Paper contributions

The contributions of this paper according to the research gaps are presented as follows:

- Stochastic and scheduling and energy management of a microgrid is implemented using the 2 m + 1 two-point estimation method (PEM) considering the mobile energy storage system (MESS) and demand response (DR) considering renewable generation and load demand uncertainties.
- A three-dimensional multi-objective framework for stochastic scheduling and energy management of the energy microgrid is defined to minimize the operation and emission costs as well as maximize the hosting capacity
- The DR and MESS are applied in microgrid scheduling and energy management to overcome the uncertainty of renewable energy sources and increase the hosting capacity.
- A multi-objective enhanced grey wolf optimizer (MOEGWO) is proposed based on a Logistic chaotic mapping technique for circumventing local optima and achieving more precise solutions.

### Paper structure

The structure of the sections in this paper is as follows. Formulation of the problem, which includes the MESS model, the goal functions, and the constraints are presented. Then, the multi-objective optimization framework used by the problem-solver based on the MOEGWO algorithm is outlined. In this study, 2 m + 1 PEM approach to model the uncertainties is presented. Finally, the outcomes and findings summaries are given.

### Problem formulation

In this study, a stochastic and multi-objective optimization model for distribution microgrid scheduling IS proposed considering the DR and dynamic MESS based on the two-point estimation method (2 m + 1 PEM) and MOEGWO with objective of maximizing the generation hosting of ERs, minimization of operational costs as well as pollution emission cost minimization. In the following, the modeling of PV and WT is presented along with the energy storage.

### Microgrid model

A microgrid includes distributed generation and renewable energy sources, energy storage, and load demand that can operate independently of or connected to the main power grid. The microgrid studied in this research is a 33-bus distribution network type and includes PV, WT, MT, FC, and dynamic MESS.

*Wind energy model:* The WT's production power is calculated using manufacturer data and wind speed information. The generated electricity of a wind turbine is defined by the following formula^2,4,5^.

1 $$P_{WT}^{{}} = \left\{ \begin{gathered} 0\begin{array}{*{20}c} {} & {} & {} \\ \end{array} \,;v_{W} \le v_{cut\,in} ,\,v_{W} \ge v_{cut\,out} \hfill \\ P_{WT,\max } .\left( {\frac{{v_{W} - v_{cut\,in} }}{{v_{rated} - v_{cut\,in} }}} \right)^{m} ;v_{cut\,in} \le v_{W} \le v_{rated} \hfill \\ P_{WT,\max } + \hfill \\ \frac{{P_{furl} - P_{WT,\max } }}{{v_{cut\,out} - v_{rated} }}.\left( {v_{W} - v_{rated} } \right)\,;v_{rated} \le v_{W} \le v_{furl} \hfill \\ \end{gathered} \right.$$

where P_WT_ is wind turbine output power, *v*_*W*_ wind speed, *v*_*cutin*_ low cut-off speed, *v*_*cutout*_ cut-out wind speed in m/s, *P*_*WT,max*_ is upper turbine output power in kW and *P*_*furl*_ is output power at high cut-out wind speed.

To model the uncertainty of wind power, the most appropriate distribution is the Weibull PDF, therefore, in this study, the Weibull PDF is used for the wind speed, and it should be evaluated by considering the appropriate parameters of the wind speed change curve. This function can be shown as follows^4^:

2 $${f}_{x}(x)=\frac{\beta w}{\eta }.(\frac{x}{\eta }{)}^{\beta w-1}.{e}^{-(\frac{x}{\beta w}{)}^{\beta w}}$$

where x is the wind speed, η and $\beta w$ are the scale parameter and the shape parameter, respectively.

*PV energy model:* The production power of the PV panel is calculated based on the manufacturer's data and radiation and temperature data. The output power of a PV panel is defined as follows^2,6^.

3 $${P}_{PV}(t)={P}_{rated}.(\frac{s}{{s}_{ref}}).{\eta }_{MPPT}$$

where P_rated_ is the rated power of the photovoltaic panel, S_ref_ solar radiation, reference solar radiation (1000 W/m^2^) and ${\eta }_{MPPT}$ is tracking efficiency of the maximum photovoltaic power point (in this study, it is considered equal to 0.95)^2,6^.

According to the behavior of solar radiation, beta PDF is applied to model it according to the following equation^34^.

4 $${f}_{b}(s)=\left\{\begin{array}{l}\frac{\Gamma (\alpha s+\beta s)}{\Gamma (\alpha s)\Gamma (\beta s)}.{s}^{(\alpha s-1)}.(1-s{)}^{(\beta s-1)}\;for\;0\le s\le 1,\alpha s,\beta s\ge 0\\ 0otherwise\end{array}\right.$$

where s is the solar radiation in kilowatts per square meter, f_b_(s) is the statistical density function of the beta distribution for the variable s, and $\alpha s$ and $\beta s$ are parameters of the beta distribution are calculated as follows.

5 $$\beta s=(1-\mu e).(\frac{\mu e.(1+\mu e)}{\sigma {e}^{2}}-1)$$

6 $$\alpha s=\frac{\mu e.\beta s}{1-\mu e}$$

where $\mu e$ and $\sigma e$ are the mean and standard deviation of this distribution, respectively.

*Dynamic MESS model:* The dynamic MESS is comparable to conventional energy storage systems^2^, which at first are typically employed to provide reserve energy for unreliable energy generation in the power distribution network. The distinction between traditional energy storage and dynamic energy storage is the fact that dynamic MESS can be transferred based on the system's objective functions or particular conditions, such as system reconfiguration or a rise in production capacity. Dynamic MESS acts as a load or generator according to the state of charge and discharge. Energy management decides whether or not to receive energy from the dynamic MESS during system operation.

7 $$\left\{ {\begin{array}{*{20}c} {SOE \left( 0 \right) = SOE \left( T \right)} \\ {SOC \left( T \right) = \frac{{SOE{ }\left( t \right)}}{{E_{MESS} }}} \\ {E_{MESS} = \frac{{SOE{ }_{max} { }{-}{ }SOE_{min} }}{{SOC_{max} - { }SOC_{min} }}} \\ {SOC \left( t \right) = SOC \left( {{ }t - 1{ }} \right) + P_{MESS}^{ch} \left( t \right). \eta_{MESS}^{ch} - \frac{{P_{MESS}^{dch} \left( t \right)}}{{\eta_{MESS}^{dch} }}} \\ \end{array} } \right.$$

where SOE is available energy of the MESS, SOC denotes state of charge of the MESS. The SOC is ratio of available energy to the maximum available capacity of the MESS. E_MESS_ is the MESS maximum available capacity, SOC_min_ and SOC_max_ refer to the lower and upper SOC of the MESS, SOE_min_ and SOE_max_ are lower and upper SOE of the MESS. SOE (t-1) represents the charging state of the storage at hour t-1, ${P}_{MESS}^{ch}(t)$ and ${P}_{MESS}^{dch}(t)$ refer to the MESS charge and discharge power and ${\eta }_{MESS}^{ch}$ and ${\eta }_{MESS}^{dch}$ represent MESS charging and discharging efficiency.

### DR model

The DR is considered as incentive based program.The following equations show how their behavior can be modeled. In this study, electricity consumers are divided into three categories: residential, commercial, and industrial. Constraints indicate that the total quantity of energy saved by each user throughout each hour should be less than or equal to the upper quantity of its offers^35^.

8 $$RP\left(r,t\right)=RC\left(r,t\right).{\xi }_{r,t} , RC(r,t)\le {RC}_{t}^{max}$$

9 $$CP\left(c,t\right)=CC\left(c,t\right).{\xi }_{c,t} , CC(c,t)\le {CC}_{t}^{max}$$

10 $$IP\left(i,t\right)=IC\left(i,t\right).{\xi }_{i,t} , IC\left(i,t\right)\le {IC}_{t}^{max}$$

where r, c, and i are the residential (RC), commercial (CC), and industrial (IC) consumers number; $RC(r,t)$, $CC(c,t)$, and $IC\left(i,t\right)$ denote load reduction planned amount by each RC, CC, and IC consumer in period t; ${RC}_{t}^{max}$, ${CC}_{t}^{max}$, and ${IC}_{t}^{max}$ refer to reduction of the maximum demand recommended by each consumer in period t; ${\xi }_{r,t}$,, ${\xi }_{c,t}$, and ${\xi }_{i,t}$ clear the incentive payment amount to each consumer in t; and $RP\left(r,t\right)$, $CP\left(c,t\right)$, and $IP\left(i,t\right)$ are load reduction cost by RC, CC, and IC consumers in t for the recommended reducing the demand , respectively.

### Objective function

**Figure 1:**
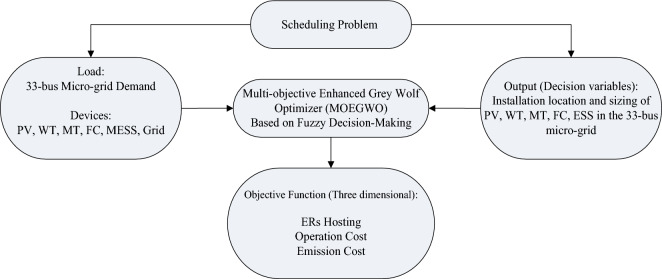
Proposed framework of stochastic and multi-objective scheduling of the microgrid.

The problem of stochastic and multi-objective scheduling of a microgrid including ERs, dynamic MESS, and DR strategy is formulated in form of a multi-objective optimization model. The stochastic and multi-objective scheduling model is presented to maximize the renewable generation hosting capacity of ERs, minimizing the operational costs as well as pollution emission cost minimization via a 2 m + 1 PEM considering ERs generation and load demand uncertainties. In addition, a DR program based on incentive-based payment has been applied to eliminate the uncertainty parameters effect. In Fig. 1, the scheduling model is depicted. The framework of the proposed scheduling model based on the MOEGWO-based fuzzy decision-making is shown in Fig. 1.

*ERs generation hosting:* The objective function of ERs generation hosting includes power generation of WT, PV, microturbine (MT) and fuel cell (FC) resources, which is defined as follows:

11 $$Max\, { F}_{1}={F}_{{P}_{DG}}=\sum_{i=1}^{{N}_{DG}}{P}_{DG} \left( i\right)$$

where ${F}_{{P}_{DG}}$ represents the function of renewable generation hosting capacity of the network of energy sources, ${N}_{DG}$ is the number of sources and ${P}_{DG}$ is the power of each energy source. Here, the objective is to maximize system revenue from microgrid electricity sales (electricity generation multiplied by unit cost is defined as revenue). Of course, according to the other objective functions that are defined in following, based on the multi-objective optimization framework and fuzzy decision making, a compromise is made between them.

*Operating cost:* The objective function of operation cost includes the cost of energy losses, the cost of purchased power from the main grid, the operation cost of ERs, the cost of storage, in addition a DR program are defined as follows^35,36^:

12 $$\begin{aligned} Min{ }F_{2} = F_{{C_{Op} }} = & \mathop \sum \limits_{t = 1}^{T} P_{Loss} { }\left( {{ }t{ }} \right).C_{Loss} { } + \mathop \sum \limits_{t = 1}^{T} P_{Grid} { }\left( {{ }t{ }} \right).C_{Grid} { } + \mathop \sum \limits_{t = 1}^{T} P_{PV} { }\left( {{ }t{ }} \right).C_{PV} \\ { } & + \mathop \sum \limits_{t = 1}^{T} P_{WT} { }\left( {{ }t{ }} \right).C_{WT} + \mathop \sum \limits_{t = 1}^{T} P_{MT} { }\left( {{ }t{ }} \right).C_{MT} { } + \mathop \sum \limits_{t = 1}^{T} P_{FC} { }\left( {{ }t{ }} \right).C_{FC} { } + \mathop \sum \limits_{t = 1}^{T} P_{MS} { }\left( {{ }t{ }} \right).C_{MS} { } \\ & + \mathop \sum \limits_{t = 1}^{T} P_{DR} { }\left( {{ }t{ }} \right).C_{DR} \\ \end{aligned}$$

where ${F}_{{C}_{Op}}$ represents the operating cost function. ${P}_{Loss} \left( t\right)$, ${P}_{Grid} \left( t\right)$, ${P}_{PV} \left( t\right)$, ${P}_{WT} \left( t\right)$, ${P}_{MT} \left( t\right)$, ${P}_{FC} \left( t\right)$, ${P}_{MS} \left( t\right)$ and ${P}_{DR} \left( t\right)$ represent power loss, power purchased from the grid, , PV power, WT power, MT power, FC power, storage power and unsatisfied load demand due to the incentive package caused by DR. ${{\text{C}}}_{{\text{Loss}}}$ is the price of each kW of losses, ${{\text{C}}}_{{\text{Grid}}}$ is the price of grid electricity, ${{\text{C}}}_{{\text{PV}}}$ and ${{\text{C}}}_{{\text{WT}}}$ is the price of PV and WT electricity, ${{\text{C}}}_{{\text{MT}}}$ is the price of MT electricity, ${{\text{C}}}_{{\text{FC}}}$ is the price of FC electricity, ${{\text{C}}}_{{\text{MS}}}$ is the price of each kWh of storage power and ${{\text{C}}}_{{\text{DR}}}$ is the price of the proposed ${\text{DR}}$ package.

*Pollution emission cost:* The pollution emission formula (${F}_{{C}_{Emiss}}$) includes functions that determine the quantity of pollution produced by DG devices and by the network at the time of buying it. Pollutants consist of CO2, SO_2_, and _NOx_, and the resulting emission function is able to be obtained the following way from the pollution model^35^:

13 $$Min { F}_{3}={F}_{{C}_{Emiss}}=\sum_{t=1}^{T}{C}_{Emiss-DG} \left( t \right) +\sum_{t=1}^{T}{C}_{Emiss-Grid} \left( t \right)$$

where ${C}_{Emiss-DG} \left( t\right)$ and ${C}_{Emiss-Grid} \left( t\right)$ represent the cost of pollution caused by energy resource units and the cost of the pollution caused by the purchase of grid electricity, respectively and T is the simulation period.

The average pollution caused by ERs can be calculated as follows:

14 $${C}_{Emiss-DG} \left( t \right)=\sum_{i=1}^{{N}_{DG}}\left({C}_{Emiss-DG}^{{CO}_{2}} \left( i \right)+{C}_{Emiss-DG}^{{SO}_{2}} \left( i \right)+{C}_{Emiss-DG}^{{NO}_{x}} \left( i \right)\right).{P}_{DG,i} \left( t \right)$$

where ${C}_{Emiss-DG}^{{CO}_{2}}$, ${C}_{Emiss-DG}^{{SO}_{2}}$ and ${C}_{Emiss-DG}^{{NO}_{x}}$ are pollution coefficients of CO_2_, SO_2_ and NO_x_ due to ERs (kg/MWh) caused by DG. ${P}_{DG,i} \left( t\right)$ is the production power of the i^th^ energy source at time t.

Likewise, the pollution resulting from the grid during the procurement of energy can be expressed by

15 $${C}_{Emiss-Grid} \left( t \right)=\sum_{i=1}^{{N}_{DG}}\left({C}_{Emiss-Grid}^{{CO}_{2}} \left( i \right)+{C}_{Emiss-Grid}^{{SO}_{2}} \left( i \right)+{C}_{Emiss-Grid}^{{NO}_{x}} \left( i \right)\right).{P}_{Grid} \left( t \right)$$

where ${C}_{Emiss-Grid}^{{CO}_{2}}$, ${C}_{Emiss-Grid}^{{SO}_{2}}$ and ${C}_{Emiss-Grid}^{{NO}_{x}}$ are pollution coefficients of CO_2_, SO_2_ and NO_x_ due to grid (kg/MWh). ${P}_{Grid} \left( t \right)$ is the power purchased from the grid at time t.

### Constraints

The objective function of the microgrid scheduling problem should be optimized subjected to the following constraints^35–37^:

- Power balance
  16 $$\sum_{i=1}^{{N}_{DG}}{P}_{DG,i} \left( t \right)+ {P}_{Grid} \left( t \right)={P}_{Demand} \left( t \right)-{P}_{DR} \left( t\right)$$

where ${P}_{Demand} \left( t\right)$ and ${P}_{DR} \left( t\right)$ respectively express the power demanded by the load and the unsupplied power of the load due to the DR strategy at time t.

${P}_{DR} \left( t\right)$ is the quantity of engaged involvement in demand response strategies and is defined by

17 $${P}_{DR} \left( t \right)=\sum_{r}RC(r,t)+\sum_{c}CC(c,t)+\sum_{i}IC(i,t)$$

- ERs powerwhere ${P}_{DG,i}^{min} \left( t\right)$ and ${P}_{DG,i}^{max} \left( t\right)$ are the lower and upper power values of energy sources at time t, respectively.
  18 $${P}_{DG,i}^{min} \left( t \right)\le {P}_{DG,i} \left( t \right)\le {P}_{DG,i}^{max} \left( t\right)$$

- MESS capacitywhere Eq. (19) denotes the SOC maximum and minimum values of the MESS. ${P}_{MESS-charge}^{max} \left( t\right)$ and ${P}_{MESS-discharge}^{max} \left( t\right)$ are the MESS maximum charging and discharging power, respectively at time t. γ(t)$\in \left\{\left.\mathrm{0,1}\right\}\right.$ and γ(t) = 1 and γ(t) = 0 clear the discharge and charge modes of the MESS, respectively.
  19 $${SOC}_{min}(t)\le {SOC}_{min}(t)\le {SOC}_{min}(t)$$
  20 $${P}_{MESS}^{ch} \left( t \right) \le {P}_{MESS-charge}^{max} \left( t \right).\left( 1- \gamma \left(t\right)\right)$$
  21 $${P}_{MESS}^{dch} \left( t \right) \le {P}_{MESS-discharge}^{max} \left( t \right).\gamma \left(t\right)$$

### The stochastic model

In this study, the stochastic and multi-objective scheduling of a microgrid is performed including ERs, dynamic MESS, and also DR strategy considering uncertainties of ERs generation and load demand uncertainties, and forecasting the ERs generation hosting is one of the major challenges in microgrid scheduling researches. Uncertainty modeling methods are divided into three categories: MCS, analytical methods, and approximate methods. In this study, the PEM based on approximate methods is used to model uncertainties. The PEM, unlike the MCS, does not depend on the PDF of uncertain variables, and due to their approximateness, it can overcome these problems considering its first few statistical moments. Also, compared to the MCS, it has a lower computational cost and iteration of convergence. The 2 m + 1 PEM is derived from statistical information that utilizes the minimum estimated value (the central instances of the input randomized variables). The empirical instances of the output variables may be determined by multiplying 2 m + 1 times the objective function with solely the two middle instances of each unpredictable input variable. In the PEM, the information gathered from the central instances is applied for identifying certain indicative locations (s locations for each variable) termed centers. These representation points are used for solving the approach, and the statistical data of the uncertain output variable can be determined using the representative locations' answers^38–40^. To calculate the central instances of the output variables for the stochastic microgrid scheduling challenge, the following 2 m + 1 PEM execution phases^38–40^ have been provided:

Step 1 Specify the total amount of variable inputs (m).

Step 2 Setting the moment vector of the output variable as $E({U}^{i})=0,i=\mathrm{1,2}$.

Where, $E({U}^{i})$ represents the ith moment vector of the output variable.

Step 3 Setting $$c=1(c=\mathrm{1,2},...,m)$$.

Step 4 Two standard coordinates of the unpredictable variable are presented by

22 $${\zeta }_{c,j}=\frac{{\lambda }_{c,3}}{2}+(-1{)}^{3-j}.\sqrt{{\lambda }_{c,4}-\frac{3{\lambda }_{c,3}^{2}}{4}}j=\mathrm{1,2}$$

where ${\zeta }_{c,j}$ expresses the standard places of the random input variable, ${\lambda }_{c,3}$ is skewness of the random input variable ${z}_{c}$ and ${\lambda }_{c,4}$ is expression of the kurtosis of the random input variable ${z}_{c}$.

Step 5) The positions ${z}_{c}$ are defined as follows:

23 $${z}_{c,j}={\mu }_{{z}_{c}}+{\zeta }_{c,j}.{\sigma }_{{z}_{c}}j=\mathrm{1,2}$$

where ${z}_{c,j}$ is the positions of random input variables, ${\mu }_{{z}_{c}}$ mean of ${z}_{c}$ and ${\sigma }_{{z}_{c}}$ refers to the std of ${z}_{c}$.

Step 6) The microgrid deterministic scheduling problem is performed for two positions ${z}_{c}$.

24 $${U}_{c,j}=f({\mu }_{{z}_{1}},{\mu }_{{z}_{2}},...,{z}_{c,j},...,{\mu }_{{z}_{m}})j=\mathrm{1,2}$$

where ${U}_{c,j}$ indicates deterministic scheduling for places ${z}_{c}$.

Step 7 Two weighting factors of ${z}_{c}$ are determined.

25 $${g}_{c,j}=\frac{(-1{)}^{3-j}}{{\zeta }_{c,j}.({\zeta }_{c,1}-{\zeta }_{c,2})}j=\mathrm{1,2}$$

where ${g}_{c,j}$ represents weight factors of ${z}_{c}$.

Step 8 $E({U}^{i})$ is updated.

26 $$E({U}^{i})=E({U}^{i})+\sum\limits_{j=1}^{2}{g}_{c,j}({U}_{c,j}{)}^{i}$$

Step 9 Steps 4 to 8 (for c = c + 1) until all input random variables are considered.

Step 10 The microgrid deterministic scheduling problem is implemented according to the following variable vector of the input random.

27 $${z}_{\mu }=[{\mu }_{{z}_{1}},{\mu }_{{z}_{2}},...,{\mu }_{z,c},...,{\mu }_{{z}_{m}}]j=\mathrm{1,2}$$

where ${z}_{\mu }$ is the input random variable vector.

Step 11) The weight coefficient of the microgrid scheduling problem solved in step 10 is calculated as follows.

28 $${g}_{0}=1-\sum\limits_{c=1}^{m}\frac{1}{{\lambda }_{c,4}-{\lambda }_{c,3}^{2}}$$

where ${g}_{0}$ is the weighting factor of the scheduling problem.

Step 12) $E({U}^{i})$ is as follows.

29 $$E({U}^{i})={\sum\limits_{c=1}^{m}\sum\limits_{j=1}^{2}{g}_{c,j}[({\mu }_{{z}_{1}},{\mu }_{{z}_{2}},...,{\mu }_{c,j},...,{\mu }_{{z}_{m}})]}^{i}+{g}_{0}[U({z}_{\mu }){]}^{i}$$

Step 13) Knowledge of the statistical instances of the variable at random output, the mean ${\mu }_{U}$ and standard deviation ${\sigma }_{U}$ values are defined as follows.

30 $${\mu }_{U}=E(U);{\sigma }_{U}=\sqrt{E({U}^{2})-{\mu }_{U}^{2}}$$

where ${\mu }_{U}$ and ${\sigma }_{U}$ represent the output variable mean and standard deviation, respectively.

The probability distribution function of each output random variable is calculated according to the values ${\mu }_{U}$ and ${\sigma }_{U}$ and the Gram–Charlier method^41^.

### Multi-objective optimizer

#### Overview of the GWO

*Social hierarchy:* The GWO algorithm is an evolutionary algorithm based on the population of grey wolves, which is inspired by their hunting performance and social behavior. In the population of grey wolves, four types of wolves α, β, δ, and ω are defined, where α is the main leader of the group (the first level of leadership) and is responsible for many decisions such as hunting, resting and sleeping places, waking time, etc. The wolf β is in charge of the second level of leadership that helps wolf α in making decisions and is a suitable substitute in the event of Wolf α's death. The ω wolf is the lowest level in the wolf population and the last group allowed to eat food. Wolves other than those defined are named δ. In GWO, the best solution is algorithm α and the two best answers are β and ω and the rest of the solutions are considered δ^42^.

*Bait siege:* The grey wolf surrounds its prey while hunting. The encirclement behavior is defined as follows^42^.

31 $$\overrightarrow{D}=\left|\overrightarrow{C}.{\overrightarrow{X}}_{P}(t)-\overrightarrow{X}(t)\right|$$

32 $$\overrightarrow{X}(t+1)={\overrightarrow{X}}_{P}(t)-\overrightarrow{A}\overrightarrow{D}$$

where t represents the repetition number, $\overrightarrow{A}$ and $\overrightarrow{C}$ are the vector of coefficients, ${\overrightarrow{X}}_{P}$ is position vector of the prey, and $\overrightarrow{X}$ is vector of the position of the wolf.

The vector of coefficients is defined as follows^42^:

33 $$\overrightarrow{A}=2\overrightarrow{a}{\overrightarrow{r}}_{1}-\overrightarrow{a}$$

34 $$\overrightarrow{C}=2{\overrightarrow{r}}_{2}$$

where ${\overrightarrow{r}}_{1}$ and ${\overrightarrow{r}}_{2}$ represent random vectors in the interval [0,1] and $\overrightarrow{a}$ is vector with decreasing behavior from 2 to 0 in the process of iterations.

*Hunting:* is led by α wolves and sometimes β and δ wolves also help in hunting. They may also occasionally participate in hunting. In this phase, the three best elements are α, β, and δ wolves, which have more knowledge about the hunting area. Therefore, the three best elements are stored and the rest of the wolves (ω) must update their position based on the position of α, β, and δ wolves. This behavior is presented as follows^42^.

35 $${\overrightarrow{D}}_{\alpha }=\left|{\overrightarrow{C}}_{1}{\overrightarrow{X}}_{\alpha }-\overrightarrow{X}\right|,{\overrightarrow{D}}_{\beta }=\left|{\overrightarrow{C}}_{2}{\overrightarrow{X}}_{\beta }-\overrightarrow{X}\right|,{\overrightarrow{D}}_{\delta }=\left|{\overrightarrow{C}}_{3}{\overrightarrow{X}}_{\delta }-\overrightarrow{X}\right|$$

36 $${\overrightarrow{X}}_{1}={\overrightarrow{X}}_{\alpha }-{\overrightarrow{a}}_{1}{\overrightarrow{D}}_{\alpha },{\overrightarrow{X}}_{2}={\overrightarrow{X}}_{\beta }-{\overrightarrow{a}}_{2}{\overrightarrow{D}}_{\beta },{\overrightarrow{X}}_{3}={\overrightarrow{X}}_{\delta }-{\overrightarrow{a}}_{3}{\overrightarrow{D}}_{\delta }$$

37 $$\overrightarrow{X}(t+1)=\frac{{\overrightarrow{X}}_{1}+{\overrightarrow{X}}_{2}+{\overrightarrow{X}}_{3}}{3}$$

*Attacking prey:* The grey wolves end the hunting process by attacking and tiring the prey and by stopping the prey from moving. The value of $\overrightarrow{a}$ decreases when the wolf approaches the prey. When the random $\vec{A}$ values are in the interval [1, -1], the next position of a search agent can be anywhere between the current position and the prey position. Under the circumstances $\left| A \right| \prec 1$, wolves are forced to attack prey. The GWO algorithm allows search agents to update their position based on alpha, beta, and delta location and attack the prey^42^.

*Search for prey:* The GWO method mainly searches for alpha, beta and delta positions. The grey wolves diverge from each other and separate to search for prey and converge to attack it. In the condition of $\left| A \right| \succ 1$, the grey wolves are forced to diverge from the prey to hopefully search for a specific prey. $\vec{C}$ is a random number in the interval [0, 2] and provides random weights for the bait. $\vec{C}$ is also used for global exploration and avoidance of local GWO optima^42^.

The pseudocode of the GWO is presented in Algorithm 1.

**Algorithm 1:**
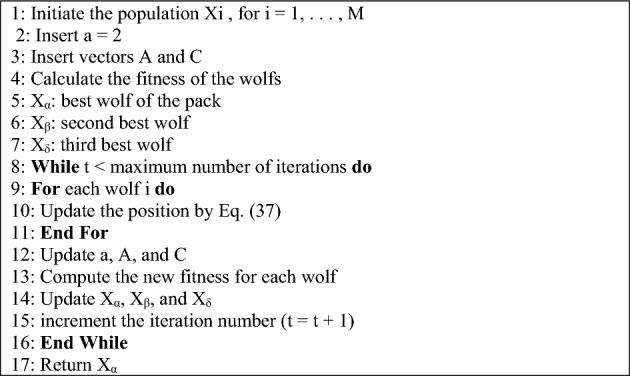
The GWO pseudo code

### Overview of the enhanced GWO (EGWO)

The GWO achieves a balance between global and local search by adjusting $\overrightarrow{A}$, thereby partially avoiding the local optimum. Nevertheless, the search process's randomization could potentially converge to a local optimum. Chaos is a prevalent nonlinear phenomenon in the natural world. It possesses the attributes of ergodicity and randomization. Therefore, chaos is frequently combined with them to enhance the global search capabilities of other optimization algorithms^43,44^. In this investigation, logistic chaos is implemented within the GWO to enhance its capability.

38 $$\begin{aligned} \chi_{i + 1} = & \kappa \chi_{i} \left( {1 - \chi_{i} } \right) \\ \chi_{i} \in \left( {0,1} \right),\chi_{i} \ne & 0.25,0.5,0.75 \\ \end{aligned}$$

where ${\chi }_{i}$, i, and k denote the iteration number and chaotic state related to the logistic equation, respectively. The logic equation becomes entirely chaotic when $\kappa =4$. By utilizing this methodology, logistic chaotic mapping (LCM) can assist the GWO in circumventing local optima and achieving more precise solutions.

Determine the chaos map that will be utilized in the subsequent iteration to modify the initial positions of the GWO in accordance with population conditions.

39 $$\left\{\begin{array}{c}{\overrightarrow{X}}_{i}(t)=(1-\alpha ){\overrightarrow{X}}_{i}(t)+\alpha {\delta }_{L}\\ {\delta }_{L}={\overrightarrow{X}}_{i}^{mi{n}_{i}{\overrightarrow{X}}_{i}^{max{\overrightarrow{X}}_{i}^{min}}}\end{array}\right.$$

where ${\overrightarrow{X}}_{i}^{max}$ and ${\overrightarrow{X}}_{i}^{min}$ refer to the ${\overrightarrow{X}}_{i}$ boundaries, $\alpha \in (\mathrm{0,1})$ denotes the space shrinkage factor.

**Figure 2:**
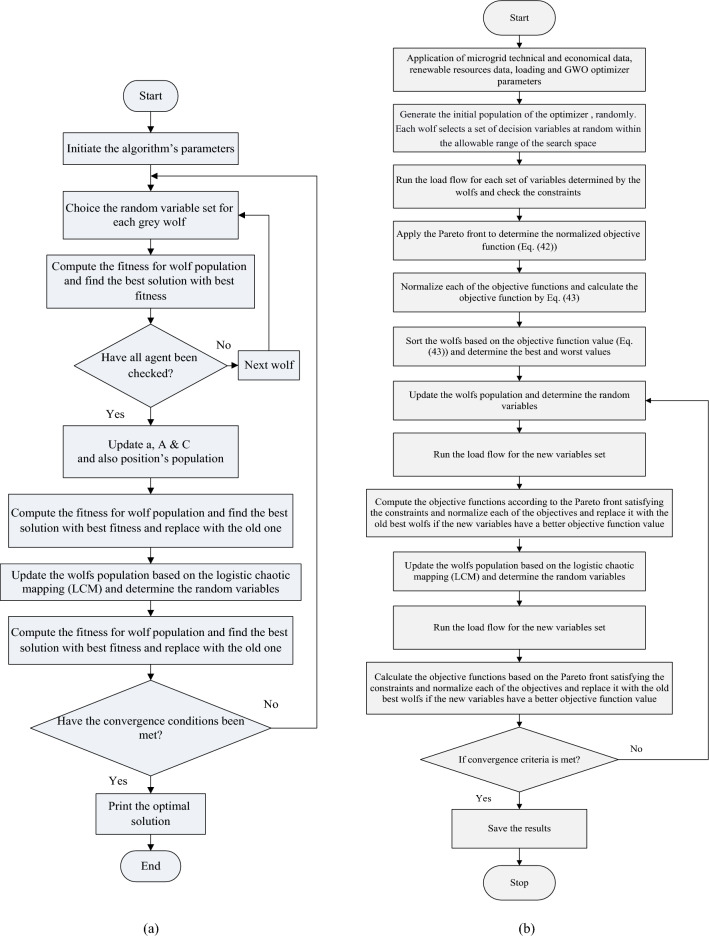
Flowchart of (**a**) EGWO (**b**) MOEGWO implementation to solve the problem.

The EGWO flowchart is shown in Fig. 2a.

### Overview of the MOEGWO

Multi-objective problem entails the concurrent optimization of multiple contradictory objectives that have to meet a variety of limitations. In single-objective optimizing, there is an optimum answer, whereas in optimization with multiple goals, there is no optimal answer, and various objectives can come into conflict. Thereby, the primary objective of solving the problem using optimization with multiple goals is to identify the Pareto front^8^ of the optimal solution to construct an acceptable compromise within every one of the goals. The multi-objective function is characterized below, taking limitations into account:

40 $$\begin{gathered} F\left( X \right) = [f_{1} \left( X \right),f_{2} \left( X \right),...,f_{z} \left( X \right)]^{T} \hfill \\ subjectedto:g\left( X \right) \le 0 \hfill \\ \end{gathered}$$

where $X$ is the decision variables vector, $F(X)$ is the decision vector objective functions, and z is the objective functions number ($z=3$).

The Pareto front consists of a number of solutions. Planners rely on intuition as a basic tool to select the final solution from among Pareto solutions. Due to the uncertainty of the planner's evaluation, the fuzzy decision-making approach is used. The membership function for zth function between kth optimal Pareto solution (${\mu }_{Z}^{k}$) is defined as follows^8^:

41 $$\mu_{{_{{_{Z} }} }}^{k} = \left\{ {\begin{array}{*{20}l} {1,} \hfill & \quad{f_{Z} (X) \le f_{Z}^{\min } } \hfill \\ {\frac{{f_{Z}^{\max } - f_{Z} }}{{f_{Z}^{\max } - f_{Z}^{\min } }},} \hfill &\quad {f_{Z}^{\min } < f_{Z} (X) < f_{Z}^{\max } } \hfill \\ {0,} \hfill & \quad{f_{Z} (X) \ge f_{Z}^{\max } } \hfill \\ \end{array} } \right.$$

where ${\mu }_{Z}$ clears the membership function value of z. ${f}_{Z}^{max}$ and ${f}_{Z}^{min}$ are the upper and lower quantities of the Zth function and ${f}_{Z}(X)$ is the *Z*th objective function quantity throughout the optimization.

${\mu }_{Z}^{k}$ clears 0 and 1 so that ${\mu }_{Z}^{k}=0$ shows the contradiction in the solution with the goals of the designer, while ${\mu }_{Z}^{k}=1$ indicates full congruence.

The normalized membership function is presented as below ^8^ for every Pareto solution k:

42 $$\mu^{k} = \frac{{\mathop \sum \nolimits_{z = 1}^{{N_{F} }} \mu_{z}^{k} }}{{\mathop \sum \nolimits_{k = 1}^{{N_{ND} }} \mathop \sum \nolimits_{z = 1}^{{N_{F} }} \mu_{z}^{k} }}$$

where ${N}_{F}$ is the non-dominant answer and ${N}_{ND}$ indicates the objective functions number.

Therefore, the compromised solution is defined by

43 $$\max \left\{ {\mu^{k} \left( X \right)} \right\}$$

The maximum value is the best compromise solution.

In this study, three objective functions (ERs generation hosting, operating cost, and pollution emission cost) are considered, and the proposed multi-objective algorithm should create a compromise between all objectives. Based on the created Pareto front solution set, the fuzzy decision-making approach selects the solution with the best compromise between three different objectives as the final solution.

### The MOEGWO implementation

In this section, the implementation steps of multi-objective and deterministic scheduling of the microgrid are described using the MOEGWO and fuzzy decision-making for minizing the operating costs, minimize the cost of pollution emission, and maximize the renewable generation hosting capacity considering DR and dynamic MESS.

Step 1 Establish data. The optimization program is currently utilizing the technical data of the microgrid, information regarding the hosting capacity of renewable generation on the ERs, the grid price, the cost of energy loss, and data regarding the operation and emission costs of renewable energy sources. Furthermore, the program has been provided with data pertaining to the normalized load demand of the microgrid over a 24-h period, in addition to the normalized power profile of PV and WT.

Step 2 Determination of the variables. At this step, for the population of grey wolves, the set of variables within the allowed range is determined, randomly. The variables are considered as installation location and size of renewable resources and also MESS in the distribution network.

Step 3 The objective function value (Eq. (42)) for each set of random variables chosen in step 2 that satisfies the operational constraints and ERs has been computed using load flow.

Step 4 Identification of the non-dominant solutions. In this stage, solutions that are not dominated by the set of solutions acquired in step 3 are identified.

Step 5 Archiving. The non-dominated solutions are segregated from the remaining solutions and archived at this stage.

Step 6 Identify the most desirable non-dominant wolf. This stage involves identifying the optimal grey wolf from the archive that was presented in step 5.

Step 7 Reconcile the population. This stage involves the update of grey wolf populations and the positions of individual members.

Step 8 The new non-dominant solution is being appended to the archive. The optimal wolf exhibiting a non-dominated solution is appended to the archive during this stage.

Step 9 Cleaning-centric solutions and superfluous components. The dominated solutions are eliminated from the clear archive at this stage. Additionally, additional archive members are removed in proportion to the total number of archive members.

Step 10 Using the logistic chaotic mapping (Eqs. (38–39)), update the population and aid the GWO in avoiding local optima in order to attain more precise solutions. This stage involves the update of grey wolf populations and the positions of individual members.

Step 11 Step 10 is considered when adding the new non-dominated solution to the archive, and the optimal wolf containing the non-dominated solution is appended to the archive. In addition to removing dominated solutions from the archive, additional members are removed in proportion to the number of archive members.

Step 12 Evaluating the criterion for convergence. The convergence criterion of the algorithm, which involves executing the utmost number of algorithm iterations, is evaluated during this phase. If convergence criteria is met, the algorithm terminates at step 13; otherwise, it returns to step 7.

Step 13 The final solution should be saved. During this stage, the final solution is determined using the fuzzy decision-making method from among the optimal solutions.

The MOEGWO implementation flowchart to solve the problem is depicted in Fig. 2b.

### The MOEGWO performance

In this section, the performance of the MOEGWO is evaluated to solve two well-known benchmark test suits, ZDT (Table 2) and CEC'09 (Table 3)^45,46^. In the ZDT test suit, MOEGWO performance is investigated to solve the six cases such as ZDT1, ZDT2, ZDT3, ZDT4, and ZDT6 as well as CEC'09 including two-objective functions (UF1-UF7) and three-objective functions (UF8-UF10). The ZDT and CEC'09 benchmark test suits are presented in detailed in^45,46^. The results of MOEGWO are compared with the MOGWO, MOPSO, MOEA/D, NSGA-II, and MOCOVIDOA. Also, for the performance metric, some indices are considered such as Inverted Generational Distance (IGD) for measuring convergence. The Spacing (SP) and Maximum Spread (MS) are employed to quantify and measure the coverage. The mathematical formulation of IGD is similar to that of Generational Distance (GD). The formulation of these indices is presented in detailed in^45,47^. All the algorithms are run 20 times on the test problems we used 300,000 function evaluations for each algorithm.

**Table 2:** Characteristics of the Zitzler-Deb-Thiele's (ZDT) benchmark functions ^45^.

Name	Objectives	Parameter domains	Characteristic
ZDT1	2	[0,1]^n^	Convex PF
ZDT2	2	[0,1]^n^	Nonconvex PF
ZDT3	2	[0,1]^n^	Many local Pareto fronts
ZDT4	2	[0,1]✕[-5,5]^n-1^	Local density solutions near
ZDT6	2	[0,1]^n^	Pareto front/nonuniformly spaced, nonconvex

**Table 3:** Characteristic of CEC'09 test functions ^46^.

Name	Objectives	Search space range	Characteristic of PF
UF1	2	[0,1]✕[-1,1]^n-1^	Concave
UF2	2	[0,1]✕[-1,1]^n-1^	Concave
UF3	2	[0,1]^n^	Concave
UF4	2	[0,1]✕[-2,2]^n-1^	Convex
UF5	2	[0,1]✕[-1,1]^n-1^	21 point front
UF6	2	[0,1]✕[-1,1]^n-1^	One isolated point and two disconnected parts
UF7	2	[0,1]✕[-1,1]^n-1^	Continuous straight line
UF8	3	[0,1]^2^✕[-2,2]^n-2^	Parabolic
UF9	3	[0,1]^2^✕[-2,2]^n-2^	Planar
UF10	3	[0,1]^2^✕[-2,2]^n-2^	Parabolic

**Figure 3:**
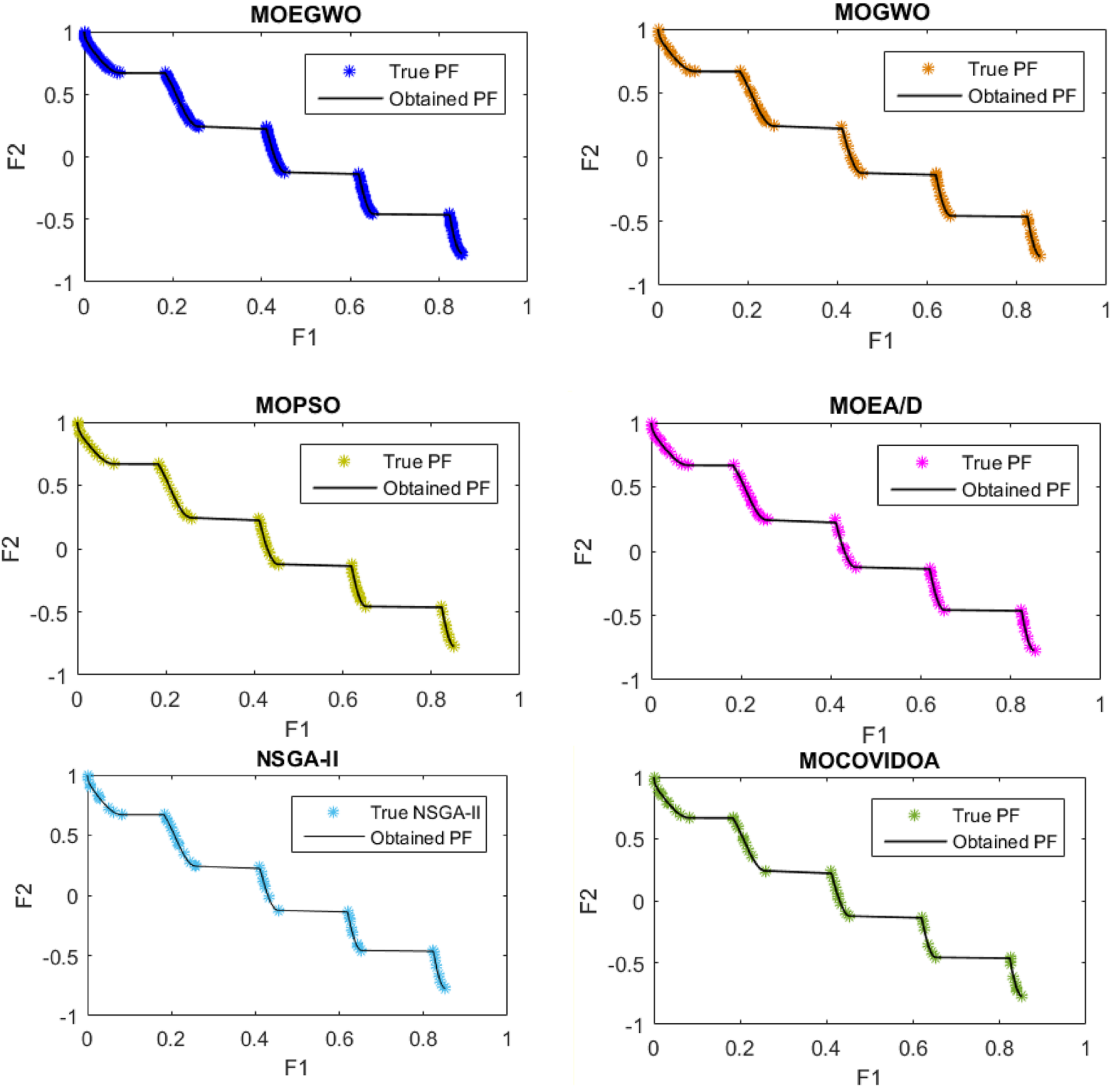
Best Pareto optimal front obtained on ZDT3 problem by MOEGWO, MOGWO, MOPSO, MOEA/D, NSGA-II, and MOCOVIDOA.

For example, the best Pareto fronts obtained from the execution of all algorithms including the proposed MOEGWO and the MOGWO, MOPSO, MOEA/D, NSGA-II and MOCOVIDOA^47^ algorithms for solving the ZDT3 test function are depicted in Fig. 3. Based on this figure, it shows the convergence and high coverage of the Pareto optimal solutions obtained by the proposed algorithm, because the obtained Pareto front is almost the same as the actual Pareto front for all cases. It can be seen that the proposed MOEGWO algorithm shows the most convergence and coverage compared to other algorithms, and its Pareto front is better than other algorithms.

The results of Table 4 show the superiority of the MOEGWO method over the MOGWO, MOPSO, MOEA/D, NSGA-II, and MOCOVIDOA algorithms in most indicators and in 20 out of 24 indicators for 6 test functions, the proposed method obtained the best results for five cases. However, in the remaining one case (ZDT6), other algorithm such as MOCOVIDOA is slightly better than the proposed method.

**Table 4:** Results for ZDT test functions.

Function	Metric	MOEGWO	MOGWO	MOPSO	MOEA/D	NSGA-II	MOCOVIDOA ^47^
ZDT1							
	IGD	**0.0031420**	0.0053735	0.0051100	0.0126900	0.0045300	0.0034000
	GD	**0.0000556**	0.0002355	0.0004414	0.0004116	0.0001543	0.0000756
	MS	**0.5839822**	0.8528273	0.9788900	1.5740000	1.6425000	0.6769100
	SP	**0.0032286**	0.0051827	0.0090021	0.0117790	0.0050178	0.0035578
ZDT2							
	IGD	**0.0033374**	0.0062849	0.0053234	0.0097974	0.0041723	0.0037042
	GD	**0.0000287**	0.0000763	0.0004322	0.0052154	0.0000833	0.0000339
	MS	**0.6284646**	0.9002664	1.0787800	1.7000000	1.5497000	0.7737700
	SP	**0.0017368**	0.0033296	0.0084532	0.0074974	0.0053305	0.0020165
ZDT3							
	IGD	**0.0025311**	0.0075673	0.0116510	0.0080097	0.0048673	0.0140560
	GD	**0.0004630**	0.0007936	0.0008908	0.0007859	0.0005052	0.0007451
	MS	**0.5736001**	0.8510024	0.7418100	0.5632800	1.1718000	0.8477900
	SP	**0.0045328**	0.0143521	0.0182750	0.0130730	0.0058912	0.0140560
ZDT4							
	IGD	**0.0106651**	0.0143563	0.0342531	0.0923300	0.0128282	0.0118080
	GD	**0.0000504**	0.0000722	0.0005441	0.0043800	0.0000617	0.0000861
	MS	**0.7297514**	0.8642673	0.9675700	1.5673000	0.8024400	0.7618900
	SP	**0.0028763**	0.0038548	0.0092780	0.0453700	0.0043051	0.0037710
ZDT6							
	IGD	0.0040428	0.0042075	0.0042184	0.0083440	0.1863300	**0.0039223**
	GD	0.0000295	0.0000410	0.0035230	0.0033820	0.0192260	**0.0000263**
	MS	0.9898350	1.1560831	0.9212300	1.4356000	0.9858100	**0.9666700**
	SP	0.0054932	0.0064492	0.0077384	0.0097600	0.0070768	**0.0051128**

Significance values are in bold.

**Figure 4:**
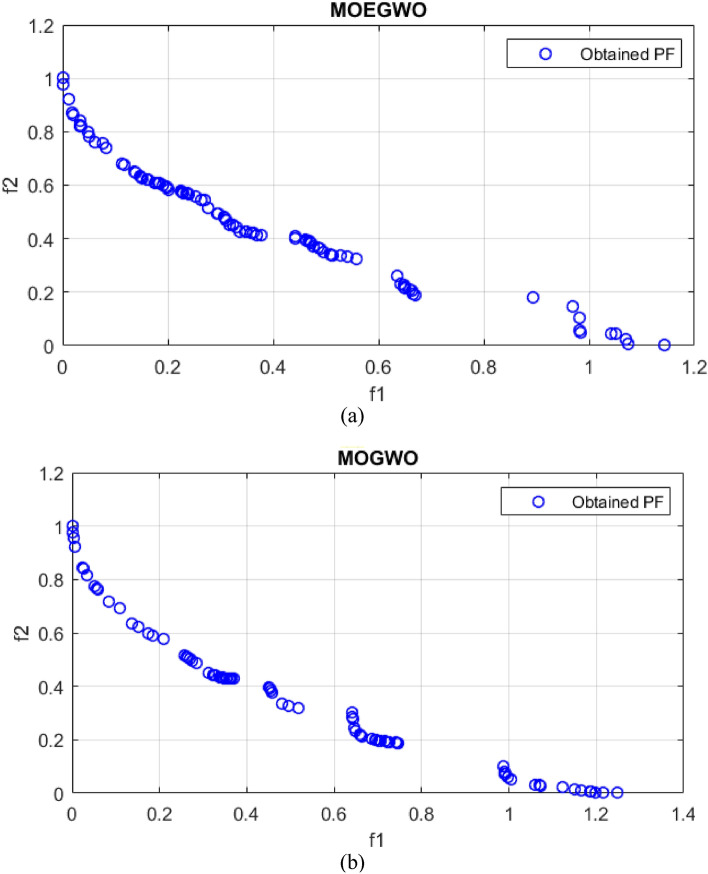
Best Pareto optimal front obtained on UF2 problem (**a**) MOEGWO (**b**) MOGWO.

**Figure 5:**
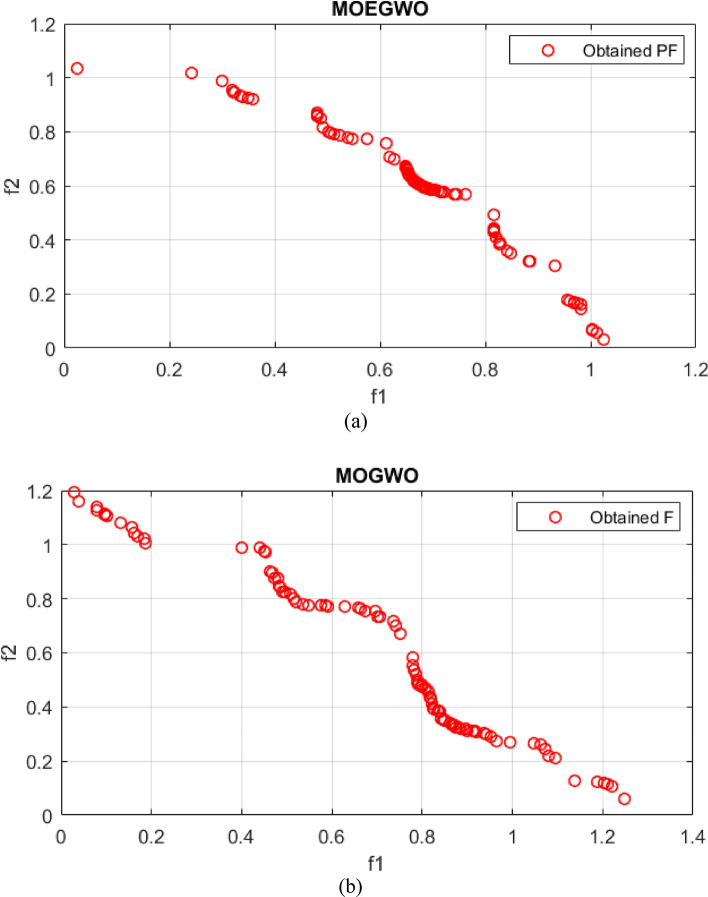
Best Pareto optimal front obtained on UF4 problem (**a**) MOEGWO (**b**) MOGWO.

**Figure 6:**
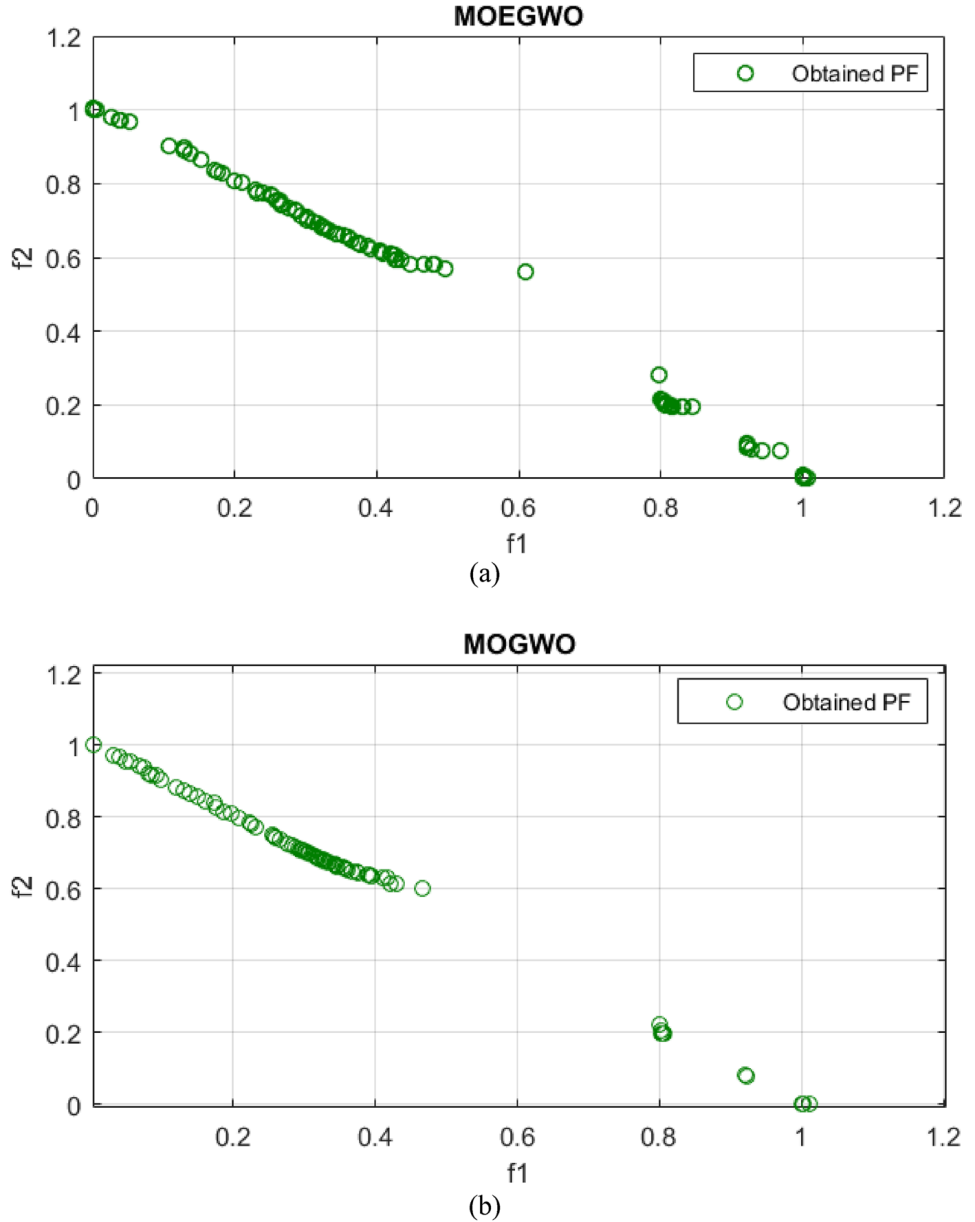
Best Pareto optimal front obtained on UF7 problem (**a**) MOEGWO (**b**) MOGWO.

The real Pareto optimal front and the best Pareto optimal front for UF2, UF4 and UF7 obtained from the MOGWO algorithm and its improved version, MOEGWO, are shown in Figs. 4, 5, 6. It can be seen that the optimal Pareto front of MOEGWO is better than MOGWO in the implemented tests and the coverage of the Pareto front is wider. Also, the results show that MOEGWO Pareto optimal solutions have better distribution in both objectives.

As shown in Table 5, the MOEGWO in 8 functions UF1, UF2, UF3, UF5, UF6, UF8, UF9 and UF10 has obtained the best results in all evaluation criteria compared to other algorithms. For functions UF4 and UF7, the MOCOVIDOA^47^ has obtained better criteria than other algorithms. Therefore, in most functions, the proposed MOEGWO has shown better performance.

**Table 5:** Results for CEC'09 test functions.

Function	Metric	MOEGWO	MOGWO	MOPSO	MOEA/D	NSGA-II	MOCOVIDOA ^47^
UF1							
	IGD	**0.0470293**	0.0683170	0.0634330	0.2171600	0.0807200	0.0541270
	GD	**0.0037922**	0.0062330	0.0085620	0.0097130	0.0086960	0.0044620
	MS	**0.7563583**	0.9792352	1.3510100	1.2280000	1.6470000	0.8309000
	SP	**0.0082176**	0.0621600	0.0637380	0.0072095	0.0076305	0.0105930
UF2							
	IGD	**0.0328428**	0.0381703	0.0575220	0.0516600	0.0500130	0.0350020
	GD	**0.0037524**	0.0048226	0.0112530	0.0046049	0.0648800	0.0042994
	MS	**1.0744060**	1.2767923	1.2218800	1.2933000	1.3131000	1.1118000
	SP	**0.0026936**	0.0074296	0.0442810	0.0062903	0.0154310	0.0034600
UF3							
	IGD	**0.1744562**	0.1998735	0.2119200	0.4449300	0.2873400	0.1935100
	GD	**0.0210078**	0.0260352	0.0235320	0.0445060	0.0276410	0.0230520
	MS	**1.1554210**	1.2689737	1.4230200	1.7093000	1.3013000	1.2244000
	SP	**0.0173115**	0.0270516	0.0265100	0.2203700	0.0283180	0.0202600
UF4							
	IGD	0.0596772	0.0704730	0.0655230	0.0995160	0.1174000	**0.0585620**
	GD	0.0034408	0.0048340	0.0242190	0.0077704	0.0112550	**0.0032200**
	MS	0.6810622	0.7859355	0.9781100	1.4574000	1.3238000	**0.6644300**
	SP	0.0120267	0.0141252	0.0281500	0.0121850	0.0175020	**0.0111750**
UF5							
	IGD	**0.1783456**	0.2766781	0.1982020	0.6830500	0.1909900	0.1909200
	GD	**0.1272175**	0.1477392	0.1926160	0.1573590	0.1436620	0.1428100
	MS	**0.7521481**	0.9601356	1.2741300	1.0067000	1.1284000	0.8194000
	SP	**0.0152743**	0.0178731	0.2127700	0.0461900	0.0503410	0.0176000
UF6							
	IGD	**0.2529865**	0.2975627	0.3651700	0.3078700	0.7387800	0.2864000
	GD	**0.1730425**	0.2014936	0.3371300	0.3550100	0.2350100	0.1985400
	MS	**0.5477061**	0.7033479	1.3537100	1.5783000	1.2372000	0.6060400
	SP	**0.3748623**	0.3821538	0.3661700	0.4225000	0.1315700	0.3998400
UF7							
	IGD	0.0432572	0.0469927	0.0453110	0.2670000	0.0827680	**0.0428340**
	GD	0.0023806	0.0031205	0.0077820	0.0151700	0.0065911	**0.0021517**
	MS	0.0361295	0.0471064	1.2261400	1.0317000	1.4710000	**0.0352180**
	SP	1.0037803	1.0060107	0.0431200	0.0082483	0.0077955	**1.0013000**
UF8							
	IGD	**0.0876083**	0.1154822	0.1338200	0.1329000	0.2435200	0.1053600
	GD	**0.0281581**	0.0496278	0.1828100	0.0322580	0.0867320	0.0307100
	MS	**0.7091004**	0.8301217	0.7863800	1.4588000	0.8273000	0.7746300
	SP	**0.1498872**	0.1685590	0.1293700	0.1483580	0.1986300	0.1639200
UF9							
	IGD	**0.0892337**	0.1146526	0.1489100	0.1196000	0.2167900	0.1093300
	GD	**0.0530164**	0.0697320	0.2239200	0.0841910	0.0623140	0.0613920
	MS	**0.8854710**	1.1028869	1.0972200	1.4761000	1.4985000	1.0107000
	SP	**0.0253932**	0.0314241	0.2757400	0.0322340	0.2587200	0.0275440
UF10							
	IGD	**0.1989473**	0.2165282	0.2395900	0.2370000	0.3739300	0.2175400
	GD	**0.1447620**	0.1676964	0.3624300	0.1585670	0.1882200	0.1584000
	MS	**1.1590371**	1.1833403	1.0253000	1.5162000	1.5676000	1.1770000
	SP	**0.0924479**	0.1170047	1.3943000	0.0577390	0.2247800	0.1079900

Significance values are in bold.

## Simulation results and discussion

### System data

**Figure 7:**
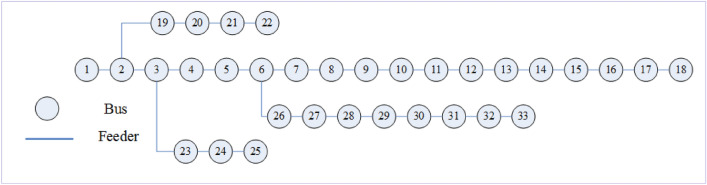
The studied 33-bus distribution microgrid.

To investigate the capability of the recommended methodology, the stochastic and multi-objective scheduling of the microgrid is performed on a 33-bus distribution microgrid that includes MT, WT, PV, FC and dynamic MESS. The 33-bus distribution microgrid is depicted in Fig. 7. The load demand of consumers is supplied through PVs, WTs, MTs and FCs or power purchased from the post. Dynamic MESSs are capable to inject energy into the grid, as well as moving during the study period, in different busses of the grid, along with each of the ERs. The microgrid lines data is taken from Ref.^42^ and load data of the modified distribution microgrid is given in Appendix A.

**Figure 8:**
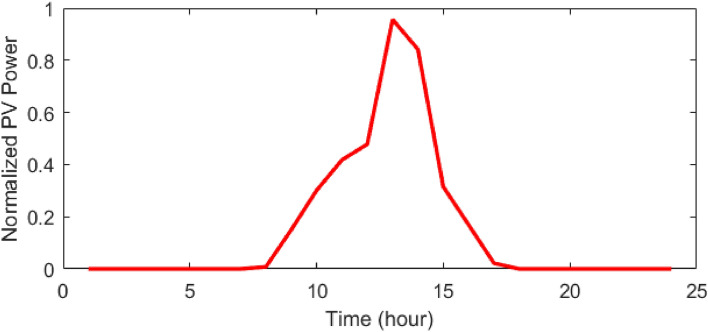
Forecasted PV power during 24 h^48^.

**Figure 9:**
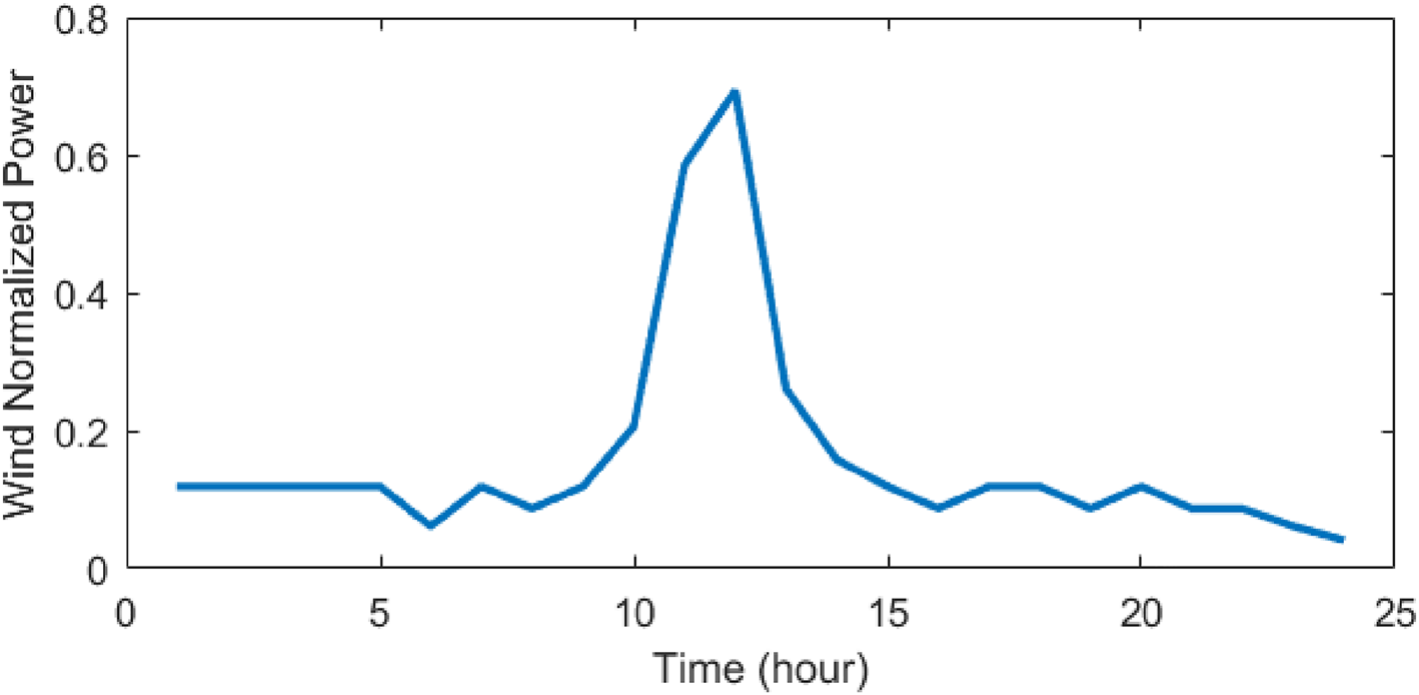
Forecasted WT power during 24 h^48^.

**Figure 10:**
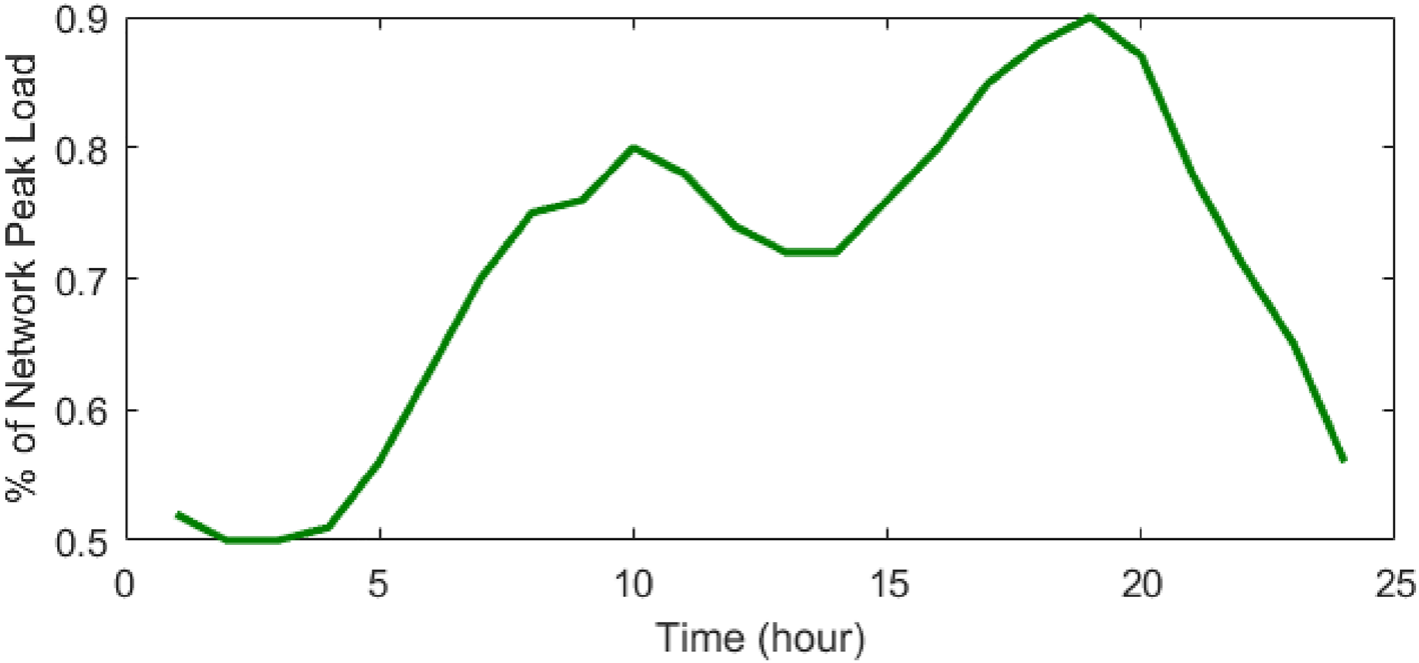
Percentage of network peak load during 24 h^48^.

**Figure 11:**
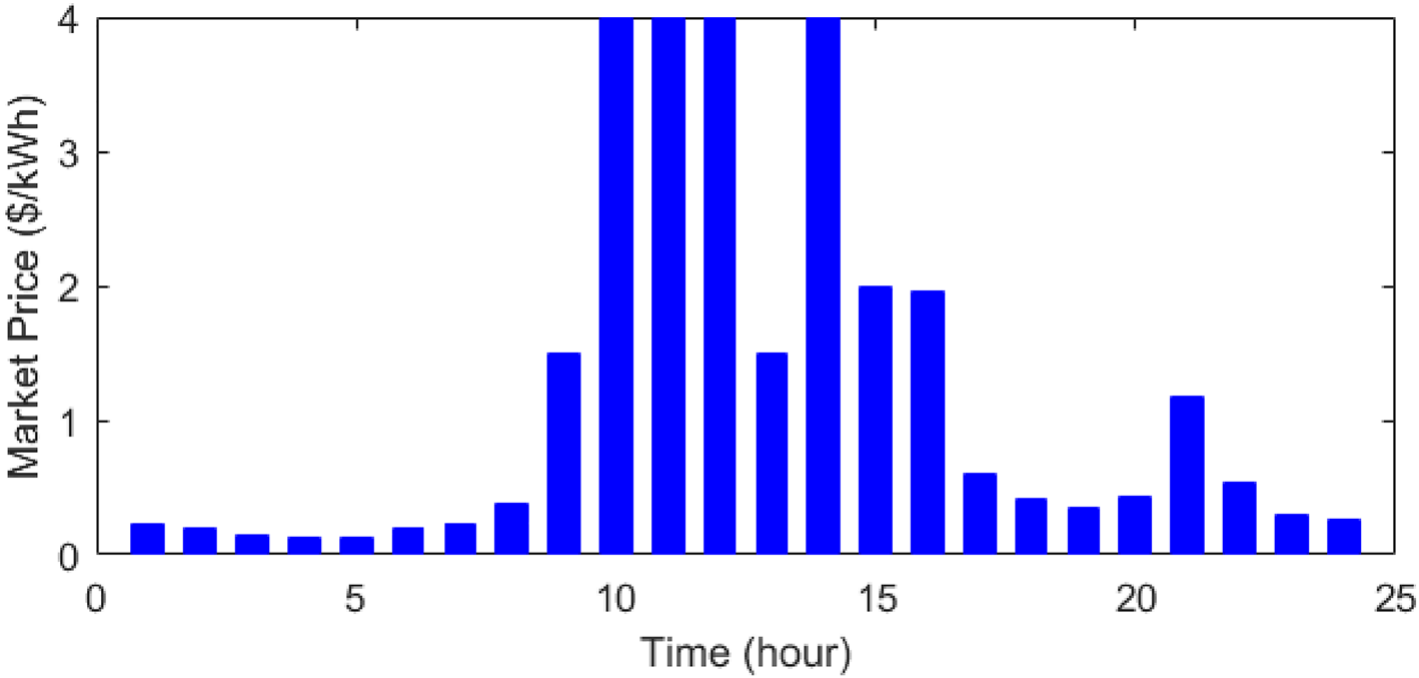
The cost of purchasing power from the network^48^.

In this study, the 2 m + 1 PEM approach is used for the stochastic microgrid scheduling. The forecasted hourly power of the PV unit and WT unit, the percentage of the peak load of the network during 24 h, and the grid price are presented in Figs. 8, 9, 10, 11^48^, respectively. Also, the electricity price and emission coefficients of different ERs and the proposed package for DR are presented in Tables 6 and 7, respectively.

**Table 6:** Electricity price and emission coefficients of different ERs ^35^.

Unit	Type	Bid ($/kWh)	CO_2_ (kg/MWh)	SO_2_ (kg/MWh)	NO_2_ (kg/MWh)	P_min_ (kW)	P_max_ (kW)
1	MT	0.457	720	0.0036	0.1	0	200
2	FC	0.294	460	0.003	0.0075	0	200
3	PV	2.584	0	0	0	0	200
4	WT	1.073	0	0	0	0	200
5	MESS	0.38	10	0.0002	0.001	− 200	200
6	Grid	–	950	0.5	2.1

**Table 7:** Recommended price-quantity offer package for DR ^35^.

Quantity (kW)	0–5	5–20	20–30	30–60
Price ($/kWh)	0.04	0.07	0.28	0.43

The proposed methodology is coded in the MATLAB 2020b software environment and by a personal computer with Corei7 specifications, with 8 GB of memory and HDD with a capacity of 1 TB. The number of population, maximum iteration, and independent executions of the algorithm is considered to be 70, 100, and 20, respectively. The effectiveness of the proposed methodology has been simulated and checked in the following cases:

- Case 1 Without considering DR and uncertainty and with static MESS
- Case 2 Without considering DR and uncertainty and with dynamic MESS (dynamic MESS effect)
- Case 3 Without considering DR and with uncertainty and dynamic MESS (effect of uncertainty)
- Case 4 Considering DR, uncertainty, and dynamic MESS (effect of DR)
- Case 5 Comparison of single and multi-objective scheduling results

## Results of dynamic MESS effect

**Figure 12:**
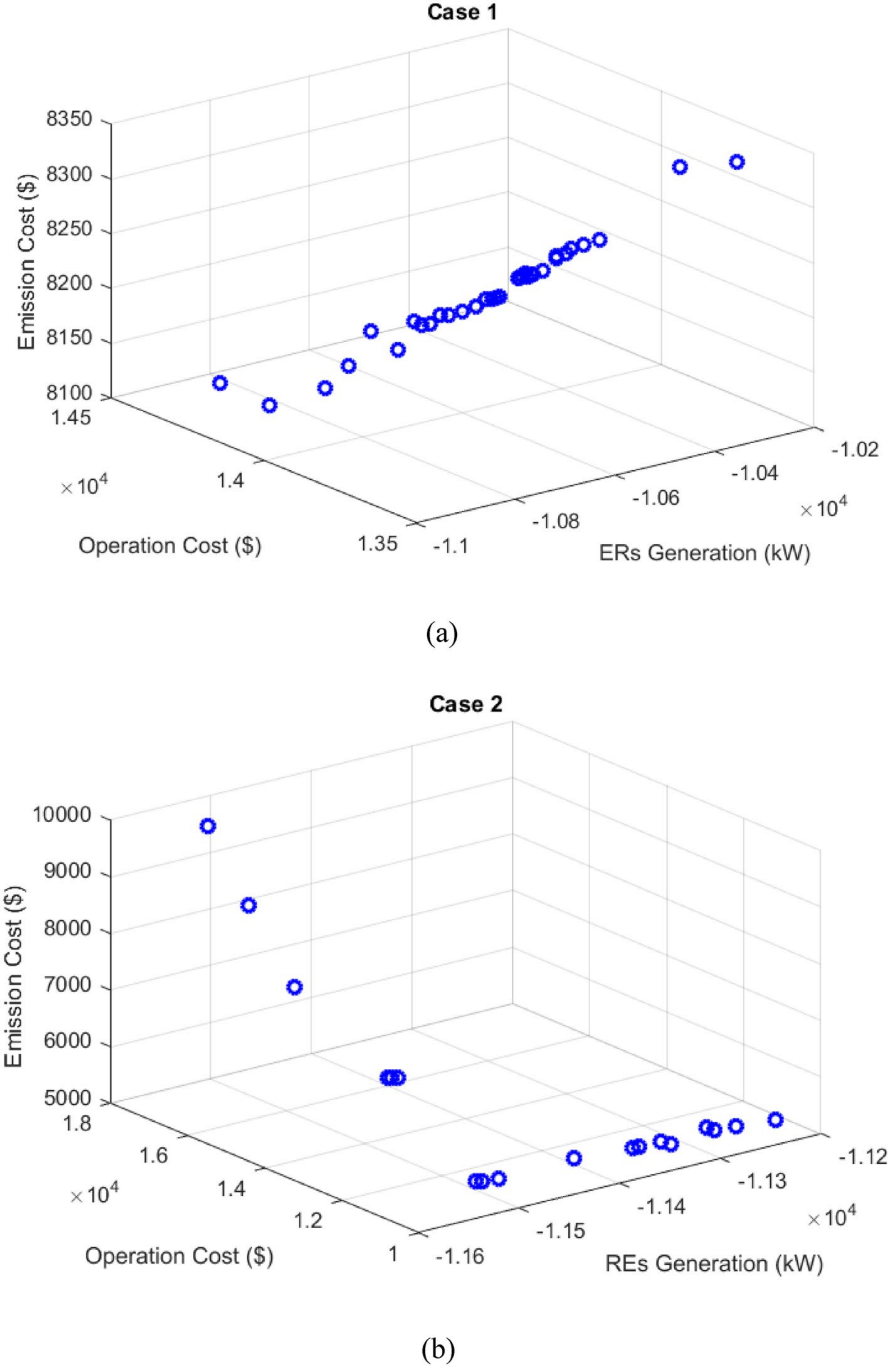
Pareto optimal solution set for (**a**) case 1 and (**b**) case 2.

In cases 1 and 2, the results of deterministic and multi-objective scheduling of microgrid with the objective of maximizing the ERs generation, minimizing operation costs, and also minimizing the cost of pollution emission, considering static and dynamic MESS, respectively using the MOEGWO. In this case, the effect of considering dynamic MESS is evaluated compared to the static MESS in solving the deterministic microgrid scheduling problem. The Pareto optimal solution set obtained for cases 1 and 2 using the MOEGWO is shown in Figs. 12a and 8b, respectively. According to Figs. 8a and 12b, it can be seen that based on the Pareto solution set, case 2 has more dispersion than case 1. It should be noted that in Fig. 8, the reason for its negative ERs is that the objective F_1_ defined in Eq. (11) is maximization, which is presented in the form of a minimizing (-F_1_) in the MATLAB coding environment.

**Figure 13:**
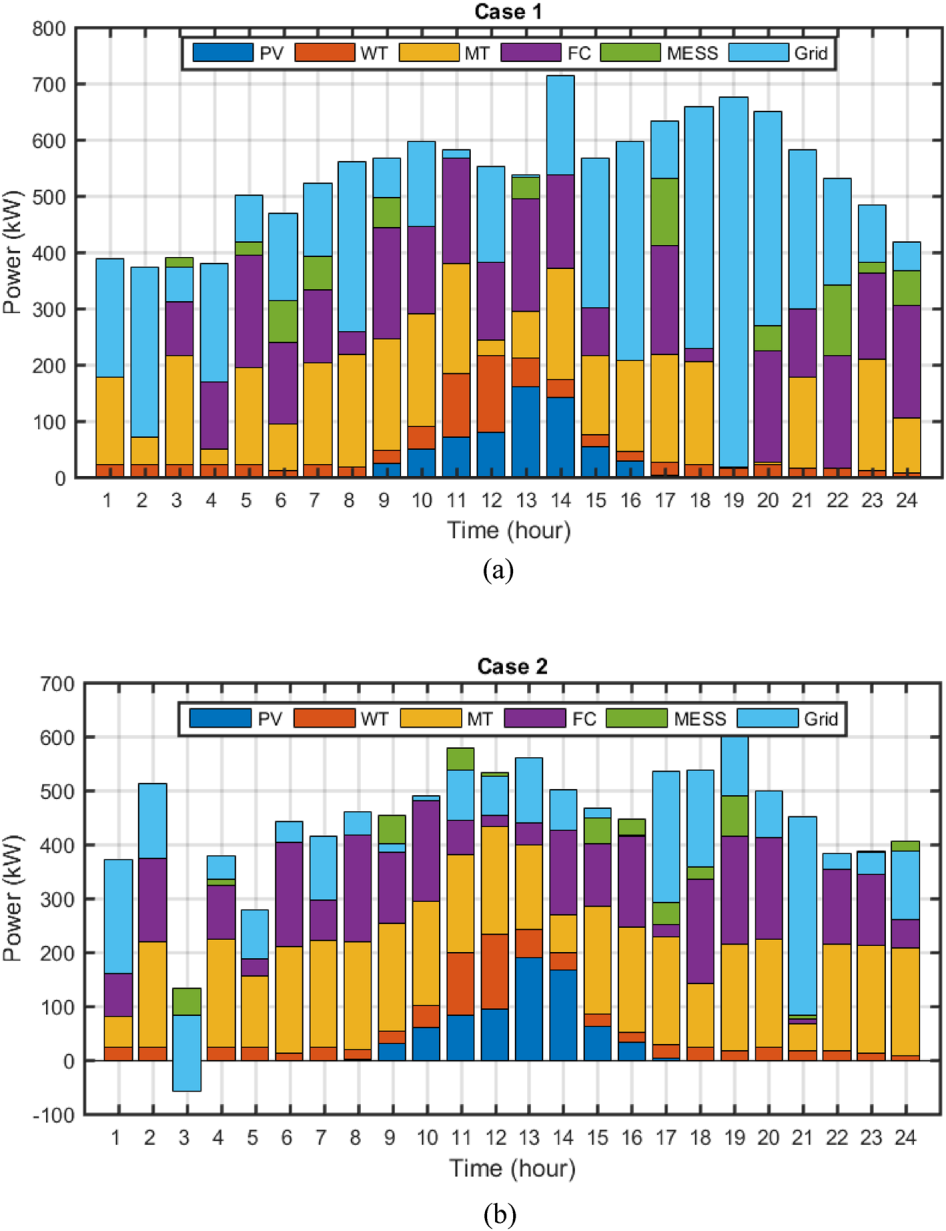
The best solution obtained for (**a**) case 1 and (**b**) case 2.

The final solution including optimal size of microgrid devices among the non-dominated solutions based on the fuzzy decision-making method is presented in Fig. 13. In these figures, the optimal and scheduled capacity of each device in the day-ahead has been determined using the MOEGWO with the aim of achieving the best performance of the microgrid.

**Figure 14:**
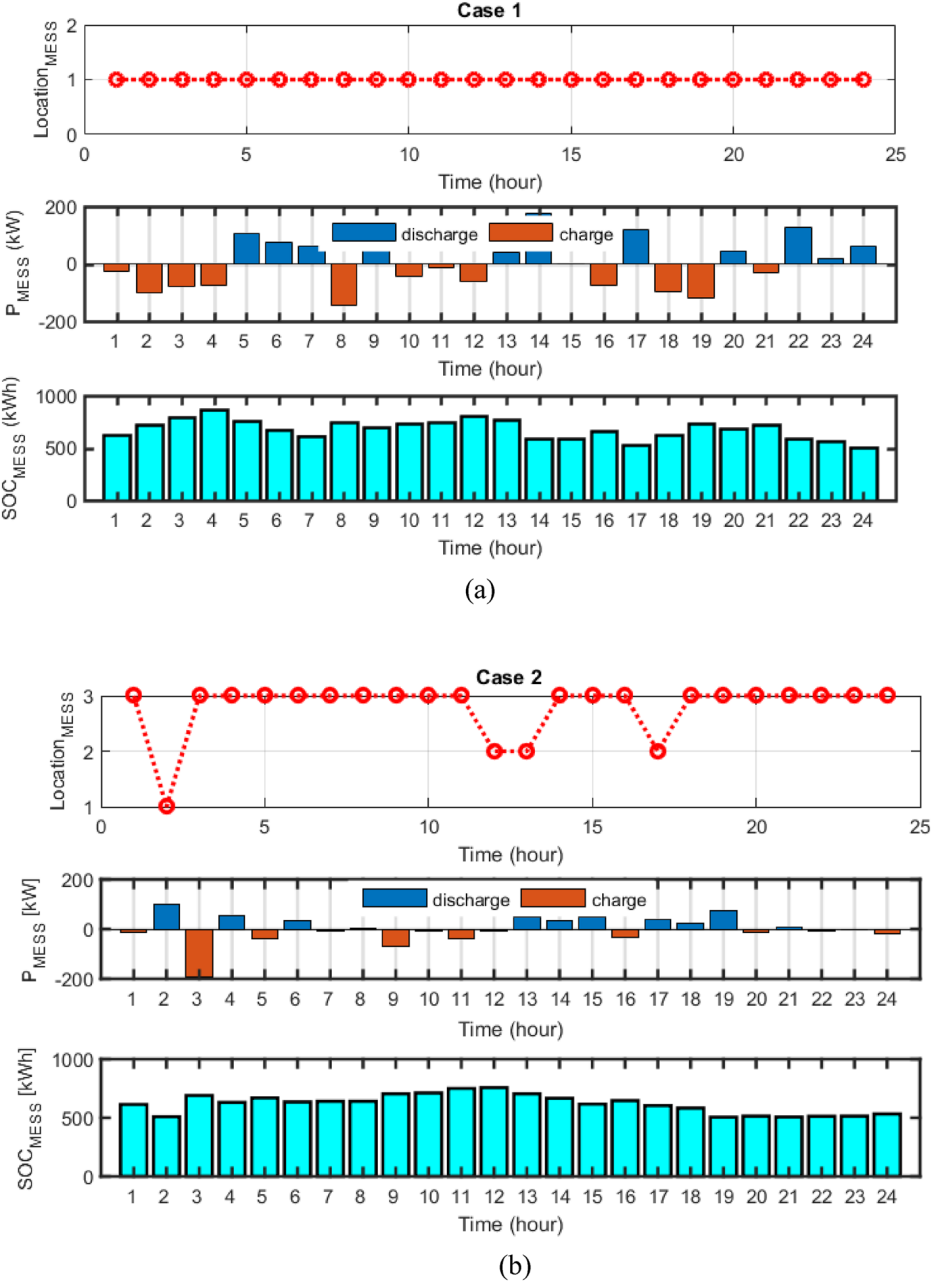
(**a**) MESS displacement scheduling, MESS power and SOC changes for a) case 1 and b) case 2.

The numerical results of multi-objective deterministic microgrid scheduling, including the installation location of each of the ERs and MESS, the peak power produced by the ERs, the cost of each device, as well as the value of the objective are given in Tables 8 and 9, respectively. Based on Tables 8 and 9, the results show that 868 kWh of battery is installed in bus 25 in case 1 with static MESS, and 756 kWh of dynamic battery MESS is shifted between buses 10, 25, and 33 during the study period. In case 1, the static storage (not portable) is located in the microgrid and it is installed in only one place (bus) in the network. The optimization algorithm installed 868 kWh of static battery storage in bus 25 of the network.On the other hand, in Case 2, the storage device is modeled dynamically and portable among the electrical network buses. The optimization algorithm has transferred the amount of 756 kWh of dynamic storage (MESS) during 24 h a day between buses 10, 25 and 33 of the electric network. The changes of this transmission during 24 h are shown in Fig. 14b. According to Table 10, by considering the dynamic MESS, the value of each objective has been improved more compared with the static MESS. Moreover, considering MESS dynamic, the cost of energy loss is reduced from $1,241,506 to $1,144,926, and the cost of network energy is decreased from $3,465,866 to $-285,632 (receiving power from the microgrid by the main grid), the cost of pollution is declined from $8,199,544 to $5,347,756 and the operation cost has been reduced from $13,938.79 to $10,685.55. Therefore, microgrid scheduling based on dynamic MESS has been able to change the location based on the objective functions of the system or increase the renewable generation hosting capacity of the network and has improved each of the objectives more compared to the scheduling based on the static MESS. Based on Tables 8 and 9, it can be seen that the amount of PV power capacity by using dynamic MESS has arised from 169 kW (in static MESS, case 1) to 198 kW (case 2), and the WT power capacity has increased from 196 to 200 kW. Also, the amount of storage capacity has decreased from 868 kWh with static MESS to 756 kWh hours with dynamic MESS, which by reducing the cost of MESS by shifting it in the network buses has improved the objectives more compared to using static MESS. The implementation of portable storage devices (Table 8) results in enhanced distribution network characteristics. As a consequence, the implementation of dynamic MESS has increased the hosting capacity for renewable resource generation and, as shown in Table 9, has decreased the costs associated with energy loss, network energy, operation, and pollution emissions.

**Table 8:** The results of energy resources scheduling for Cases 1 and 2.

Device	PV	WT	MESS	MT	FC
Case 1					
Size (kW)	169	196	868	200	200
Location (Bus)	7	12	25	9	5
Cost ($)	3310.529	2696.864	667.220	1475.301	1081.501
Case 2					
Size (kW)	198	200	756	200	200
Location (Bus)	32	33	25,10,33	11	29
Cost ($)	3884.335	2754.915	343.348	1835.025	1008.628

**Table 9:** The techno-economic scheduling results for Cases 1 and 2.

Item/Scenario	Case 1	Case 2
Ploss before (kW)	91.615	91.615
Qloss before (kVAr)	62.041	62.041
Ploss after (kW)	56.690	52.280
Qloss after (kVAr)	40.335	37.274
Cost of energy loss ($)	1241.506	1144.926
Cost of energy grid ($)	3465.866	− 285.632
Cost of energy emissions ($)	8199.544	5347.756
ERs generation (kW)	10,701.357	11,516.794
Cost of operation ($)	13,938.79	10,685.55

**Table 10:** The results of energy resources scheduling for Cases 2 and 3.

Device	PV	WT	MESS	MT	FC
Case 2					
Size (kW)	198	200	756	200	200
Location (Bus)	32	33	25,10,33	11	29
Cost ($)	3884.335	2754.915	343.348	1835.025	1008.628
Case 3					
Size (kW)	177	190	803	173	189
Location (Bus)	18	26	21,25,29	18	21
Cost ($)	3474.75	2626.48	424.88	1442.16	981.28

Changes in MESS displacement timing, MESS power, and SOC are illustrated in Fig. 14 for cases 1 and 2. The MESS remains constant, as illustrated in Fig. 10a. However, in case 2 (Fig. 14b), the dynamic MESS is transferred between network buses at 2:00, 12:00, 13:00, and 17:00. The utilization of dynamic MESS-based microgrid scheduling has resulted in a greater capacity for hosting renewable generation (Table 8), in addition to enhancing each of the objectives outlined in Table 9. Therefore, the utilization of portable storage devices results in an enhancement of network performance as a consequence of the optimal injection of scheduled energy.

## Results of uncertainty effect

**Figure 15:**
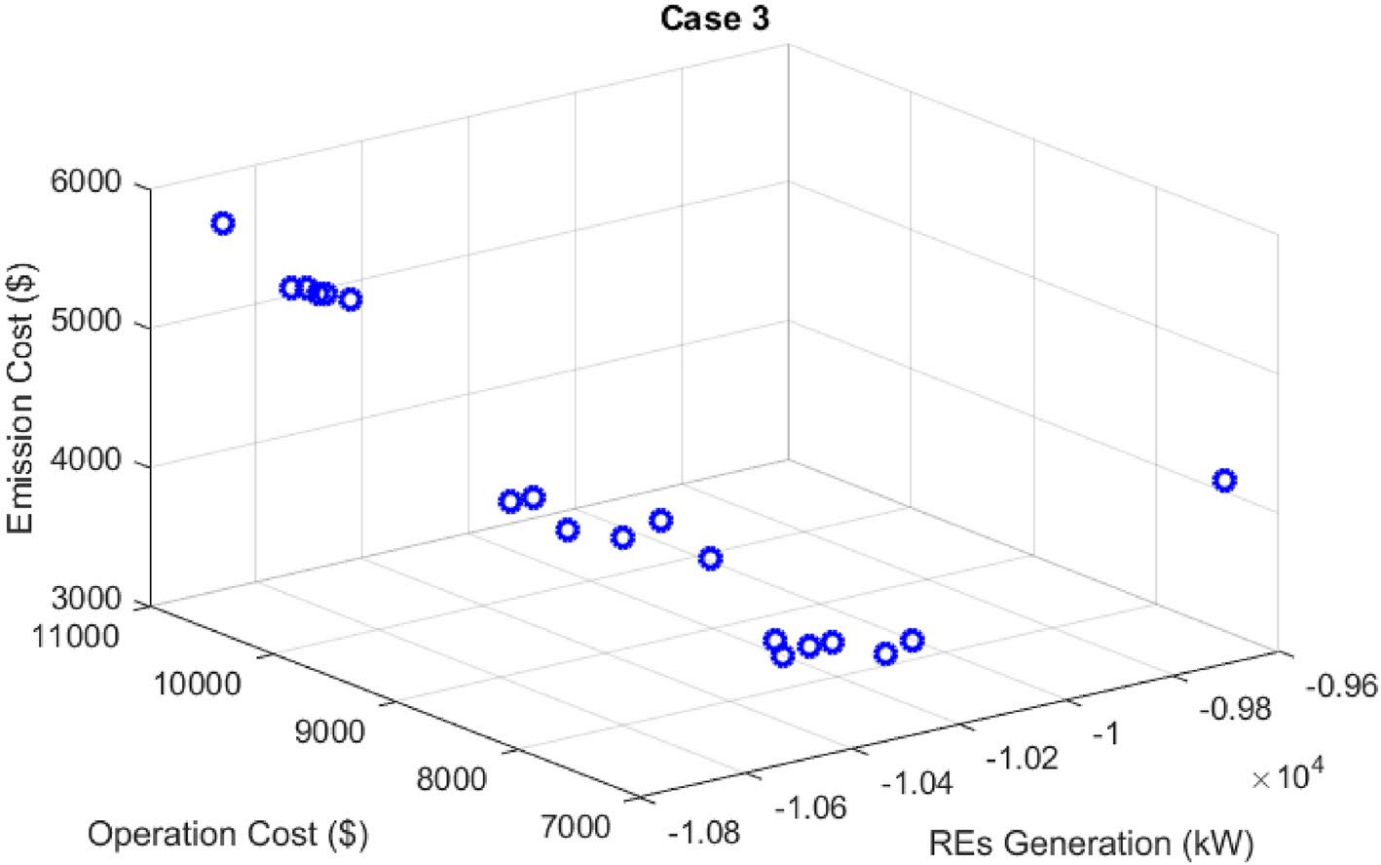
Pareto optimal solution set for case 3.

In this case, the stochastic scheduling of the distribution microgrid based on the 2 m + 1 PEM method has been implemented, considering uncertainties of the PVs and WTs generation, in addition to the load demand of the microgrid. It is assumed that the PVs and WTs generation as well as the microgrid load demand has beta, Weibull, and normal PDFs, respectively. In this section, the effect of PVs and WTs generation and microgrid demand uncertainties is investigated in solving the MOEGWO-based microgrid scheduling problem in form of a multi-objective optimization framework based on fuzzy decision-making. In this way, microgrid multi-objective scheduling has been solved using the PEM method (Case 3) due to uncertainties and its results have been compared with Case 2 (without uncertainty). In this section, the scheduling problem is implemented using dynamic MESS and considering the uncertainty and without DR. The Pareto optimal solution set for Case 3 using the MOEGWO method is shown in Fig. 15. According to Fig. 15, based on the compromise created between all objectives, the set of fuzzy solutions are distributed considering different objectives.

**Figure 16:**
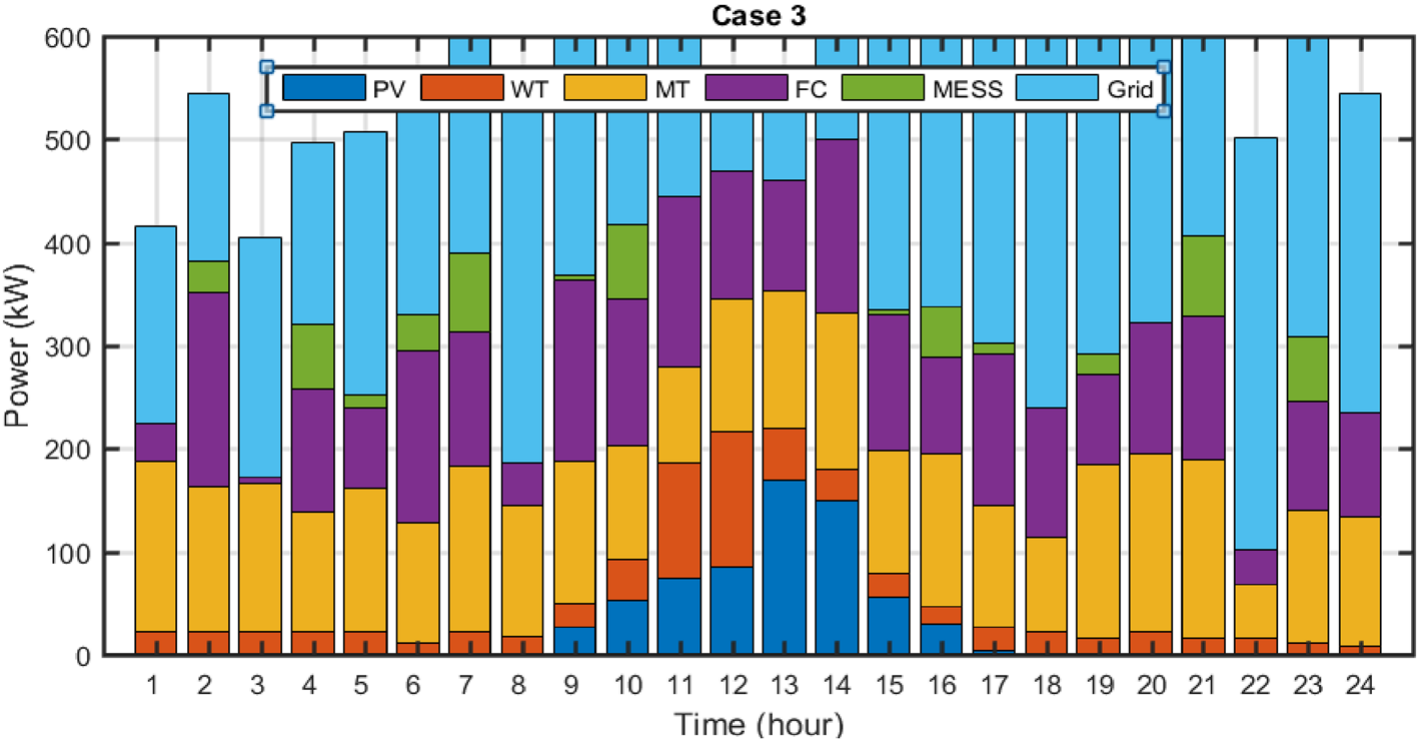
The best solution obtained for case 3.

The final solution related to the optimal size of energy sources and MESS among the non-dominated solutions based on the fuzzy decision-making method using the MOEGWO with the aim of achieving the best performance of the microgrid is shown in Fig. 16.

The tables that contain the numerical outcomes of stochastic and multi-objective microgrid scheduling are Tables 10 and 11, respectively. These tables detail the installation location and scheduled capacity of the ERs, the cost of REs, and the values of the various objectives for cases 2 and 3. According to Table 10, taking into account the uncertainties, the energy storage level has increased from 756 to 803 kWh, which is due to the uncertainties caused by the resource capacity and network load demand. Also, according to Table 11, it is clear that considering the uncertainty of operation and the emission costs has increased, and on the other hand, it has caused a decrease in the hosting capacity of renewable resources.

**Table 11:** The techno-economic scheduling results for Cases 2 and 3.

Item/scenario	Case 2	Case 3
Ploss before (kW)	91.615	91.615
Qloss before (kVAr)	62.041	62.041
Ploss after (kW)	52.280	76.11
Qloss after (kVAr)	37.274	53.43
Cost of energy Loss ($)	1144.926	1666.83
Cost of energy grid ($)	− 285.632	6620.49
Cost of energy emissions ($)	5347.756	10,444.99
ERs generation (kW)	11,516.794	10,285.95
Cost of operation ($)	10,685.55	17,236.91

**Figure 17:**
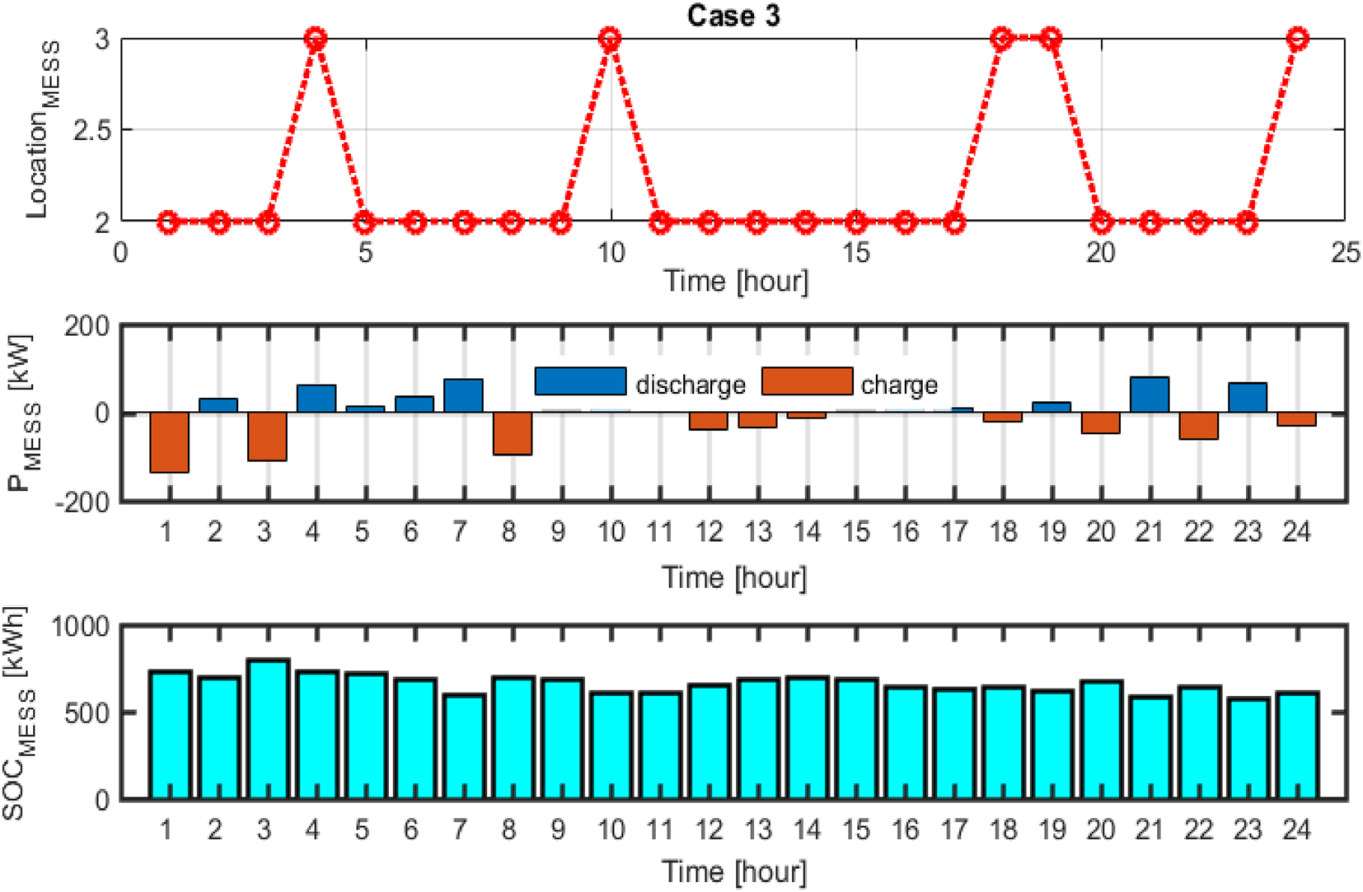
MESS displacement timing, MESS power and SOC changes for Case 3.

The dynamic MESS displacement scheduling pattern, power capacity, and their SOC changes for case 3 are presented in Fig. 17. It can be seen that the dynamic MESS is shifted between the network buses at 4:00, 10:00, 18:00, 19:00, and 24:00. The results demonstrated that the number of dynamic MESS displacements increased compared to the deterministic scheduling and without uncertainty in network buses, and on the other hand, the reserve power level increased to compensate the uncertainties caused by resource power.

## Results of DR effect

**Figure 18:**
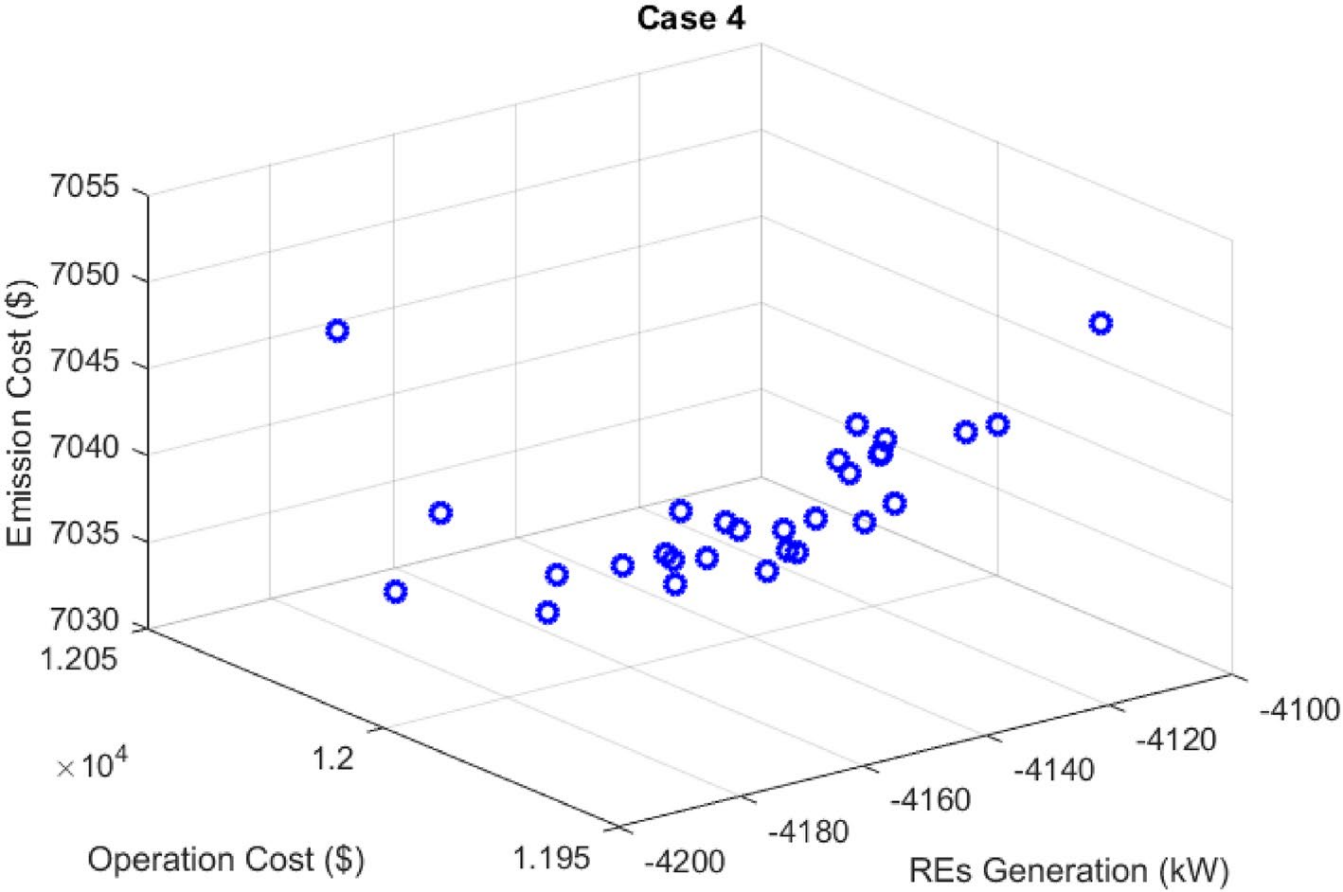
Pareto optimal solution set for case 4.

The effect of including DR in the solution of the multi-objective scheduling problem using the fuzzy decision-making method MOEGWO is presented. In this section for Case 4, the scheduling problem is implemented considering dynamic MESS, uncertainty, and DR. Figure 18 depicts the Pareto optimal solution set for Case 4 employing the MOEGWO. As depicted in Fig. 18, the set of fuzzy solutions is distributed with various objectives in mind, in accordance with the compromise reached within all objectives.

**Figure 19:**
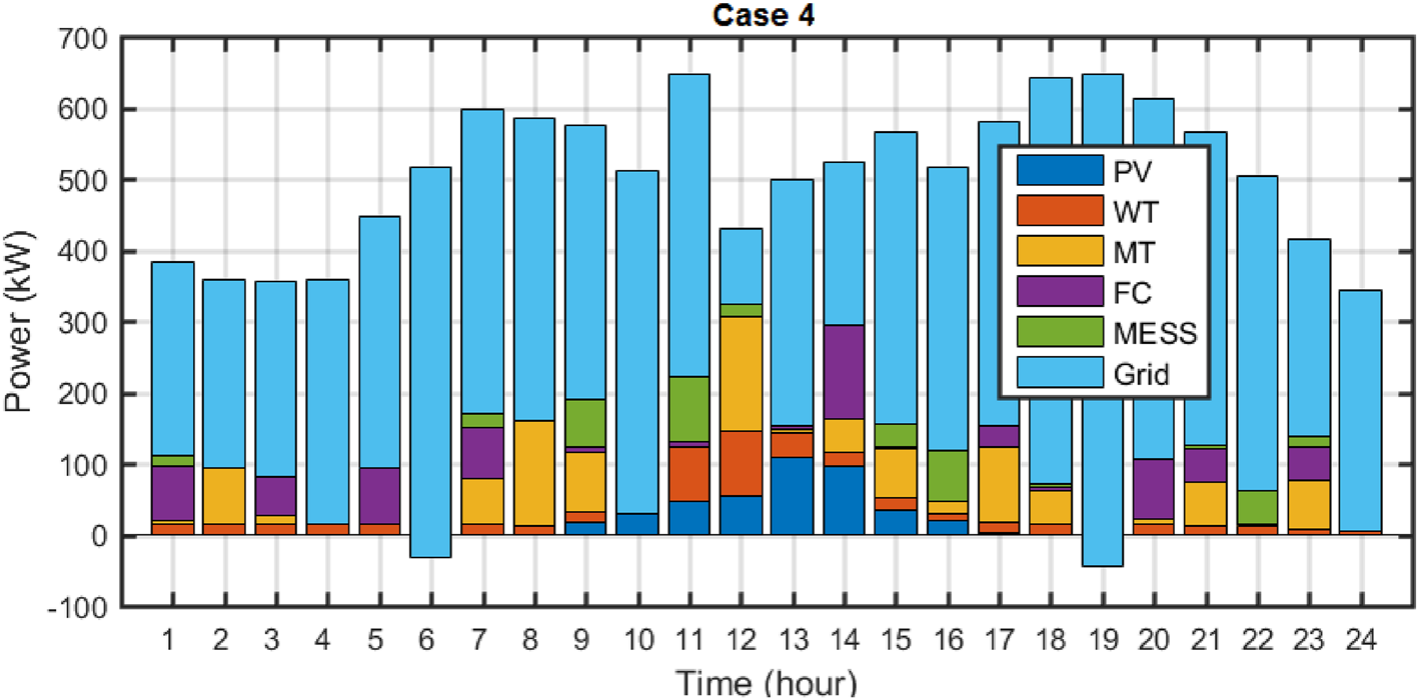
The best solution obtained for Case 4.

The final solution among the non-dominated solutions based on the fuzzy decision-making method is presented in Fig. 19. In these figure, the scheduled capacity of each microgrid device is determined optimally via the MOEGWO to obtain the best performance of the microgrid.

The numerical results of microgrid stochastic and multi-objective scheduling, including the installation location and peak capacity of the ERs, as well as the installation location and scheduled capacity of the MESS, the cost of REs, and the different objectives value are given in Tables 12 and 13, respectively. According to Tables 12 and 13, the MOEGWO in PEM-based stochastic scheduling installs 114 kW of PV power, 131 kW of PV power, 160 kW of MT power, and 152 kW of FC power in buses 6, 7, 9, and 11, respectively. It has also moved 806 kWh hours of the MESS in buses 14, 15, and 21 during the simulation period. According to Table 13, by considering the DR in solving the microgrid scheduling problem using the MOEGWO method, the values of energy loss cost, operation, and pollution emission costs are decreased and the renewable generation hosting capacity is increased compared to the scheduling without DR. In the microgrid scheduling problem, considering DR, the energy loss cost has decreased from $1666.83 to $1501.81, the pollution emission cost has declined from $10,444.99 to $9825.54, and the operation cost has reduced from $17,236.91 to $15,767.27. The results show that the cost of grid purchased energy has increased from $6620.49 to $10,139.17 when DR is considered. Also, the production of ERs has decreased from 10,285.95 kW to 4521.76 kW. Therefore, the results have shown that considering DR by reducing the level of production and paying fines to the load demand, has reduced the operating and emission pollution emission costs.

**Table 12:** The results of energy resources scheduling for Cases 3 and 4.

Device	PV	WT	MESS	MT	FC	DR
Case 3						
Size (kW)	177	190	803	173	189	
Location (Bus)	18	26	21,25,29	18	21	
Cost ($)	3474.75	2626.48	424.88	1442.16	981.28	–
Case 4						
Size (kW)	114	131	806	160	152	
Location (Bus)	6	7	14,15,21	9	11	
Cost ($)	2223.41	1812.19	330.05	464.20	281.25	15.15

**Table 13:** The techno-economic scheduling results for Cases 3 and 4.

Item/Scenario	Case 3	Case 4
Ploss before (kW)	91.615	91.615
Qloss before (kVAr)	62.041	62.041
Ploss after (kW)	76.11	68.57
Qloss after (kVAr)	53.43	46.60
Cost of energy loss ($)	1666.83	1501.81
Cost of energy grid ($)	6620.49	10,139.17
Cost of energy emissions ($)	10,444.99	9825.54
ERs generation (kW)	10,285.95	4521.76
Cost of operation ($)	17,236.91	15,767.27

The results demonstrated that utilizing DR programs to alter the electricity consumption patterns of customers, the capacity of the microgrid can be better synchronized with the renewable energy resources generation and the stored energy of the MESS. The findings also established that demand response (DR) pertains to the process of balancing power grid demand by incentivizing consumers to adjust their electricity consumption to periods of greater availability or reduced demand, commonly achieved through pricing mechanisms or financial rewards.

**Figure 20:**
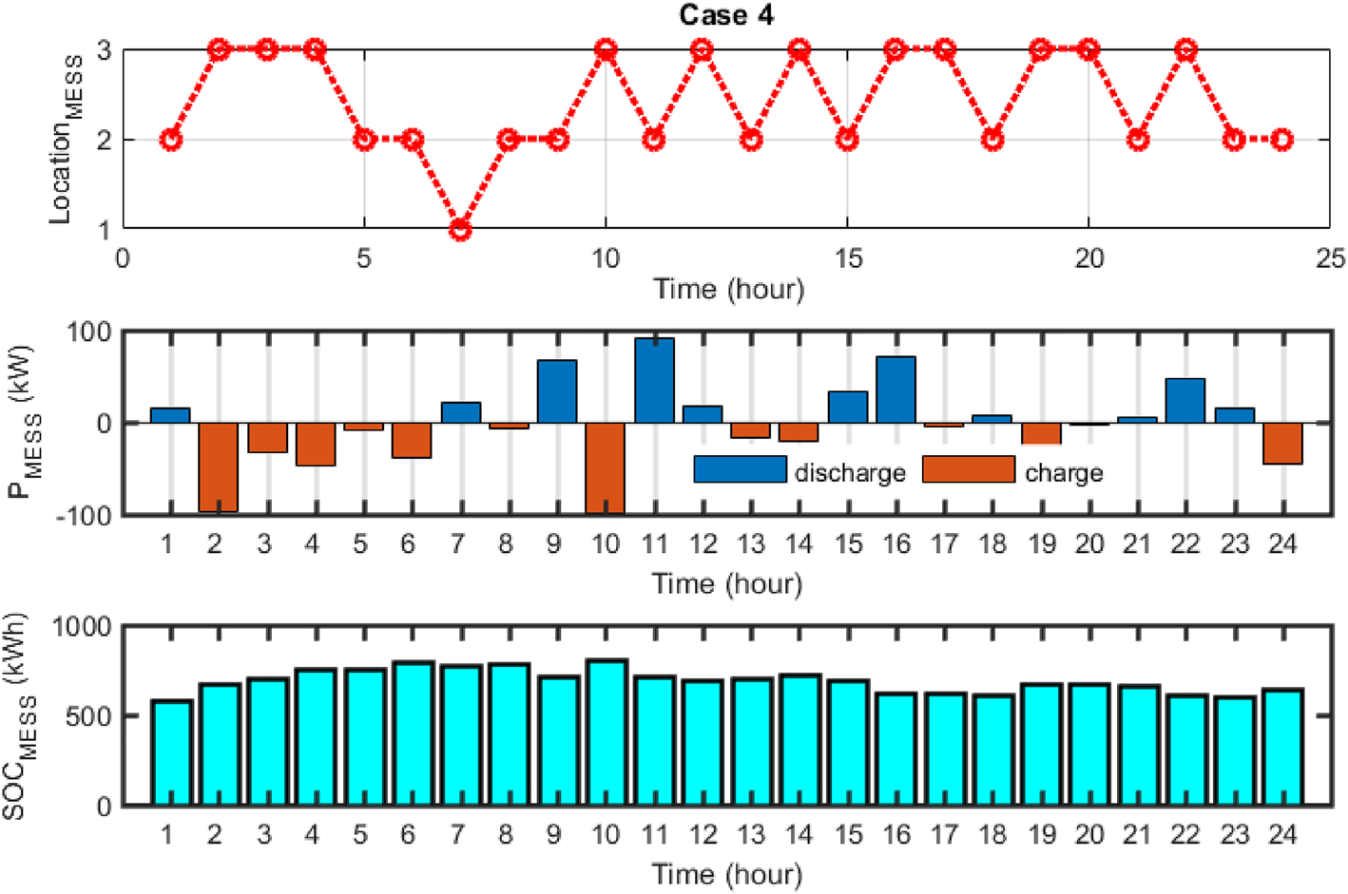
MESS displacement scheduling, MESS power and SOC changes for case 4.

In Fig. 20, the displacement timing, power and SOC changes related to the dynamic MESS for Case 4 are shown. As can be seen, in this case, the number of MESS moves is much higher than in other cases. In terms of considering DR, the program for DR has increased the number of MESS moves in the face of the proposed DR package.

## Results of multi-objective scheduling effect

The numerical results of microgrid stochastic and multi-objective scheduling, including the installation location and peak capacity of the ERs, as well as the installation location and scheduled capacity of the MESS, the cost of REs, and the value of the objects are given in Tables 14 and 15, respectively. According to Tables 14 and 15, the MOEGWO in PEM-based stochastic scheduling installs 114 kW of PV power, 131 kW of PV power, 160 kW of MT power, and 152 kW of FC power in buses 6, 7, 9, and 11, respectively. It has also moved 806 kWh hours of the MESS in buses 14, 15, and 21 during the simulation period. According to Table 15, by considering the DR in solving the microgrid scheduling problem via the MOEGWO method, the values of energy loss cost, operation, and pollution emission costs are reduced and the renewable generation hosting capacity is increased compared to the scheduling without DR. In the microgrid scheduling problem, considering DR, the energy loss cost has decreased from $1666.83 to $1501.81, the pollution emission cost has decreased from $10,444.99 to $9825.54, and the operation cost has decreased from $17,236.91 to $15,767.27. The results show that the cost of grid-purchased energy has increased from $6620.49 to $10,139.17 when DR is considered. Also, the production of ERs has decreased from 10,285.95 to 4521.76 kW. Therefore, the results have shown that considering DR by decreasing the level of production and paying fines to the load demand, has reduced the operating and emission pollution emission costs.

**Table 14:** The results of energy resources scheduling for Case 4 with different single and multi-objective function.

Device	PV	WT	MESS	MT	FC	DR
Multi-objective function (Case 4)						
Size (kW)	114	131	806	160	152	
Location (Bus)	6	7	14,15,21	9	11	
Cost ($)	2223.41	1812.19	330.05	464.20	281.25	15.15
Energy resources generation						
Size (kW)	200	154	753	200	199	
Location (Bus)	3	8	4,9,13	9	3	
Cost ($)	3915.56	2120.21	192.84	1695.29	1029.95	4.30
Operation cost minimization						
Size (kW)	23	16	852	200	155	
Location (Bus)	10	16	6,12,20	21	7	
Cost ($)	444.44	220.39	344.97	648.93	313.73	16.87
Emission cost minimization						
Size (kW)	200	117	782	158	123	
Location (Bus)	10	16	5,7,12	6	9	
Cost ($)	3915.50	1616.99	397.54	693.17	311.31	4.69

**Table 15:** The techno-economic scheduling results for Case 4 with different single and multi-objective function.

Item/scenario	Multi-objective function (Case 4)	ERs generation maximization	Operation cost minimization	Emission cost minimization
Ploss before (kW)	91.615	91.615	91.615	91.615
Qloss before (kVAr)	62.041	62.041	62.041	62.041
Ploss after (kW)	68.57	59.99	84.00	57.59
Qloss after (kVAr)	46.60	40.75	57.17	39.93
Cost of energy loss ($)	1501.81	1270.13	1839.79	1261.42
Cost of energy grid ($)	10,139.17	8555.52	11,405.80	7755.49
Cost of energy emissions ($)	9825.54	11,573.32	11,492.63	**8516.20**
ERs generation (kW)	4521.76	**10,704.14**	2864.51	5597.94
Cost of operation ($)	15,767.27	18,783.84	**14,234.95**	15,956.14

Significance values are in bold.

**Figure 21:**
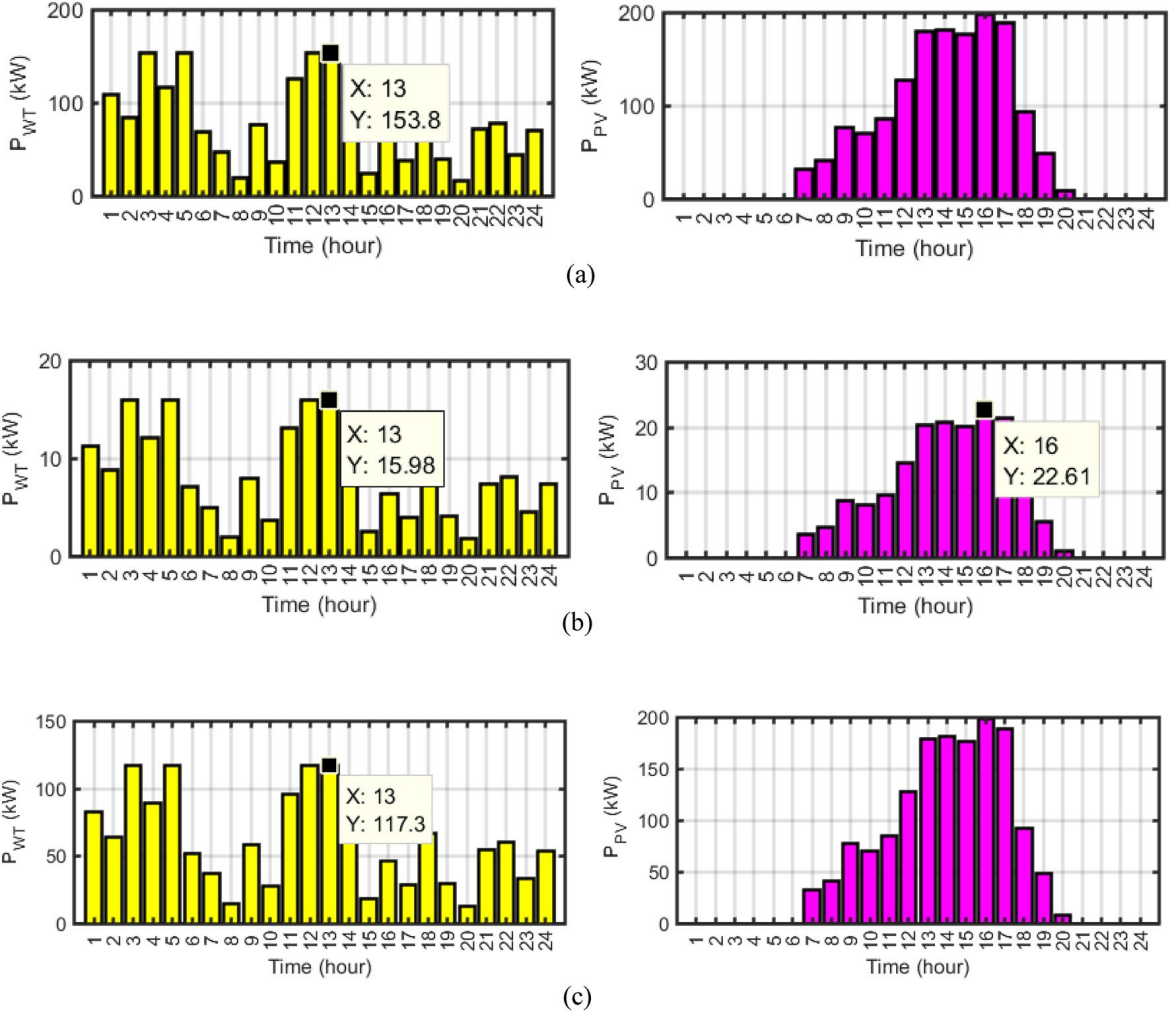
Power changes of WT and PV energy sources for (**a**) ERs generation (**b**) operation cost (b) pollution emission cost.

Power changes of WT and PV energy sources for different single-scheduling based on ERs generation, operation cost and pollution emission cost are depicted in Fig. 21. According to the figure, it can be seen that in single-objective scheduling with renewable generation hosting capacity maximization, resource generation is maximum in comparison with the other single-objective optimizations. Also, the results showed that the lowest renewable generation hosting capacity is related to the objective function of minimizing the operating cost.

## Results of MOEGWO's validation

In this section, to investigate the capability of the recommended scheduling methodology (Case 4) using the MOEGWO method, its performance is compared with the MOPSO, and MOCOVIDOA. Thus, like the MOEGWO method, the number of population, maximum iteration, and independent executions of the MOPSO, and MOCOVIDOA algorithms are considered equal to 70, 100, and 20, respectively, and the best interactive solution is considered from among the non-dominated solutions of the Pareto front set. The parameters of the traditional PSO are selected as in the reference research^49^. According to the results obtained based on the Table 16, it is clear that the MOEGWO with optimal scheduling of energy resources integrated with dynamic MESS and DR has been able to achieve lower costs of energy loss, pollution emission, and operation, as well as more renewable generation hosting capacity than the MOPSO, and MOCOVIDOA.

**Table 16:** The techno-economic scheduling results for Case 4 using MOEGWO, MOPSO, and MOCOVIDOA.

Item/Scenario	MOEGWO	MOPSO	MOCOVIDOA
Ploss before (kW)	91.615	91.615	91.615
Qloss before (kVAr)	62.041	62.041	62.041
Ploss after (kW)	68.57	75.45	77.14
Qloss after (kVAr)	46.60	52.34	54.08
Cost of energy loss ($)	1501.81	1654.50	1689.36
Cost of energy grid ($)	10,139.17	10,187.21	10,194.85
Cost of energy emissions ($)	9825.54	10,175.87	10,246.39
ERs generation (kW)	4521.76	4367.09	4351.64
Cost of operation ($)	15,767.27	15,966.38	16,032.76

By taking into account 20 independent executions, the numerical results from the MOEGWO, MOSPO, and MOCOVIDOA algorithms are reasonably compared to one another. Based on the C index (CI), the outcomes of each algorithm have been contrasted. The ability of three algorithms for solving the multi-objective optimization problem has been compared using the C index. *S*_1_ and *S*_2_ are considered to be the output Pareto solutions of two algorithms in this assessment index. The expression (*S*_1_, *S*_2_), which reflects the proportion of solutions in set *S*_2_ that are dominated by those in set *S*_1_, is defined by^50^

44 $$C({S}_{1},{S}_{2})=\frac{\left\{\left.{s}_{2}\in {S}_{2};\exists {s}_{1}\in {S}_{1}:{s}_{1}\le {s}_{2}\right\}\right.}{\left|{S}_{2}\right|}\times 100$$

where *s*_1_, *s*_2_ are the corresponding Pareto solutions for sets *S*_1_, *S*_2_, respectively. The C index is determined by the average of n independent executions, and a higher C value denotes a better Pareto solution.

In Table 17, C index is given for MOEGWO, MOSPO, and MOCOVIDOA algorithms. The results demonstrated that in rows two and three, 59.31% and 54.85% of solutions obtained by the MOEGWO dominate those achieved by MOPSO, and MOCOVIDOA in Mean value. The superior performance of the MOEGWO to obtain the better Pareto front solutions is confirmed in comparison with the MOPSO, and MOCOVIDOA.

**Table 17:** Comparison of different algorithms using the CI for Case 3 for 33-bus unbalanced network.

CI	Mean	std	Maximum	Minimum
C(MOEGWO, MOPSO)	24.08	16.33	44.78	12.66
C(MOPSO, MOEGWO)	59.31	29.04	100	0
C(MOCOVIDOA, MOEGWO)	54.85	20.11	89.63	5.37

*std denotes standard deviation.

In^51^, allocation of hybrid PV/WT/Battery system in distribution network is presented aimed active losses cost minimization, voltage profile enhancement and minimizing power purchased from the hybrid system via an improved whale optimizer algorithm (IWOA). This research is implemented for four seasons. The power loss is reduced by 15.9%, 14.94%, 11.55%, and 22.90% for summer, autumn, winter, and spring, respectively which average is a 16.32% reduction in the power losses of the 33-bus network. Based on the proposed methodology in our study, the power loss reduction of 32.28% has been obtained, which has confirmed the superior performance of the proposed method.

## Discussion

In this study, the stochastic energy management and scheduling of a microgrid with renewable energy sources and MESS was presented, with a multi-objective function that maximizes renewable generation hosting capacity and minimizes the operation and pollution emission costs considering DR and uncertainties. The stochastic approach was performed using the 2 m + 1 PEM, and the best solution was found via the MOEGWO based on a fuzzy decision-making technique. In five cases, simulations are carried out. In Case 1, microgrid scheduling results are provided without taking DR and uncertainty and with static storage. In Case 2, Case 1 was implemented, taking MESS. Case 3 presented Case 2 with the uncertainties based on the 2 m + 1 PEM. Incorporating the DR, Case 3 was implemented in Case 4. Comparing the results of single-objective and multi-objective microgrid scheduling is presented in Case 5. According to the mentioned cases, the effect of considering the MESS and uncertainties has been evaluated. Following are discussions regarding the evaluation of the outcomes derived from various cases:

- The results obtained from the comparison of cases 1 and 2 showed that microgrid scheduling based on dynamic MESS compared to static MESS leads to a greater reduction in the cost of energy losses, the cost of pollution emission, and maintenance cost, and also leads to a greater increase in the renewable generation hosting capacity the microgrid from energy resources. Therefore, the improvement achieved due to moving the dynamic storage at different hours in different buses of the distribution microgrid and optimal scheduled power injection.
- Comparison of cases 2 and 3 showed that considering the uncertainties in solving the microgrid scheduling problem using the PEM method, the values of energy loss cost, operation cost and pollution emission cost increase compared to deterministic scheduling without uncertainty. It was also found that the renewable generation hosting capacity of renewable resources has also decreased, and these changes are caused by modeled uncertainties and defined patterns in the PEM estimation method.
- Based on the comparison of cases 3 and 4, the results obtained from stochastic microgrid scheduling using the PEM and considering DR showed that the energy loss cost, operation cost and pollution emission cost were reduced compared to scheduling without the DR. Also, the generation hosting capacity of renewable resources has also decreased. Therefore, the results have shown that considering DR by decreasing the level of generation and paying fines to the load, has declined the operating costs and emission of pollution.
- In the multi-objective scheduling of the distribution microgrid, a compromise has been made between different objectives by satisfying the constraints of the microgrid and devices operation, and as a result, the capacity of energy resources is determined according to the improvement of each of the objectives of the overall objective function until the best solution was obtained among the solutions non-dominated Pareto answer set. On the other hand, in microgrid single-objective scheduling, with the significant enhancement of the objective to be optimized, some other objectives are significantly weakened.

## Conclusions and future research

This study presented a stochastic and multi-objective energy management and scheduling model of a microgrid to maximize the renewable generation hosting capacity while minimizing operation and pollution emissions costs using the 2 m + 1 PEM method and MOEGWO. The 2 m + 1 PEM was utilized for modeling the renewable generation and demand uncertainties. The simulation outcomes have been provided in evaluating the use of the MESS, examining the effect of uncertainties, and determining the effect of DR based on incentive payments.

- Among the non-dominated solutions of the Pareto front set, the multi-objective MOEGWO based on the fuzzy decision-making approach was able to determine the optimal size of the ERs and storage system.
- The simulation results showed that microgrid scheduling based on the dynamic MESS 7.8%, 34.78%, and 23.34% more reduced energy loss cost, pollution emission cost, and operation cost, respectively, as well as generation hosting capacity, has increased by 7.6% compared to the scheduling based on the static MESS.
- The results indicated that the stochastic scheduling due to the uncertainties using the 2 m + 1 PEM method causes an increase in the costs of energy losses, emission, and operation, as well as a decrease in the generation hosting capacity compared to the deterministic scheduling.
- In addition, the findings demonstrated that the implementation of the DR in solving the stochastic scheduling reduced each of the costs of energy loss, emission, and operation by 9.90%, 5.93%, and 8.535%, respectively compared to the scheduling without the DR.
- The microgrid optimization and energy management in unbalanced distribution network considering load and generation uncertainties is suggested for future work to improve the power quality indices. In this research the effect of uncertainties will be evaluated on the power quality in the network and the methods of improving the power quality will be analyzed in the conditions of uncertainties. The existing uncertainties are limitation of the research which the proposed stochastic approach able to overcome the challenges caused by the uncertainties and compensate for the resource power fluctuations by providing mobile energy storage system and demand response.

## Supplementary Information

Supplementary Information.

## Data Availability

The datasets used and/or analysed during the current study available from the corresponding author on reasonable request.

## Abbreviations

ERs: Energy resources
CC: Commercial consumer
${{\text{CC}}}_{{\text{t}}}^{{\text{max}}}$: Reduction of the maximum demand recommended by commercial consumer in period t
${{\text{C}}}_{{\text{Emiss}}-{\text{DG}}}^{{{\text{CO}}}_{2}}$: DG pollution coefficient of CO2
${{\text{C}}}_{{\text{Emiss}}-{\text{DG}}}^{{{\text{SO}}}_{2}}$: DG pollution coefficient of SO2
${{\text{C}}}_{{\text{Emiss}}-{\text{DG}}}^{{{\text{NO}}}_{{\text{x}}}}$: DG pollution coefficient of NOx
${{\text{C}}}_{{\text{Emiss}}-{\text{Grid}}}^{{{\text{CO}}}_{2}}$: Grid pollution coefficient of CO2
${{\text{C}}}_{{\text{Emiss}}-{\text{Grid}}}^{{{\text{SO}}}_{2}}$: Grid pollution coefficient of SO2
${{\text{C}}}_{{\text{Emiss}}-{\text{Grid}}}^{{{\text{NO}}}_{{\text{x}}}}$: Grid pollution coefficient of NOx
${{\text{C}}}_{{\text{Emiss}}-{\text{DG}}}$: Cost of pollution caused by energy resource units
${{\text{C}}}_{{\text{Emiss}}-{\text{Grid}}}$: Cost of the pollution caused by the purchase of grid electricity
DR: Demand response
${{\text{F}}}_{{{\text{C}}}_{{\text{Emiss}}}}$: Pollution emission function
${{\text{F}}}_{{{\text{P}}}_{{\text{DG}}}}$: Function of Ddg generation hosting
${g}_{c,j}$: Weight factors of ${z}_{c}$
IC: Industrial consumer
${{\text{IC}}}_{{\text{t}}}^{{\text{max}}}$: Reduction of the maximum demand recommended by industrial consumer in period t
MCS: Monte Carlo simulation
MESS: Mobile energy storage system
MOEGWO: Multi-objective enhanced grey wolf optimizer
MOPSO: Multi-objective particle swarm optimization
${{\text{N}}}_{{\text{DG}}}$: Number of DG sources
${N}_{F}$: Number of objective functions
${N}_{ND}$: Number of non-dominant solutions
${{\text{P}}}_{{\text{DR}}}$: Unsatisfied load demand due to the incentive package caused by DR
${{\text{P}}}_{{\text{Demand}}}$: Power demanded by the load
PDF: Probability distribution function
${{\text{P}}}_{{\text{DG}}}$: Power of each DG energy source
${{\text{P}}}_{{\text{DG}},{\text{i}}}^{{\text{max}}}$: Maximum power value of energy sources
${{\text{P}}}_{{\text{DG}},{\text{i}}}^{{\text{min}}}$: Minimum power value of energy sources
PEM: Point estimation method
${{\text{P}}}_{{\text{FC}}}$: Fuel cell power
$${{\text{P}}}_{{\text{Grid}}}$$: Grid power
$${{\text{P}}}_{{\text{Loss}}}$$: Power loss
${{\text{P}}}_{{\text{MESS}}}^{{\text{ch}}}$: MESS charge power
${{\text{P}}}_{{\text{MESS}}}^{{\text{dch}}}$: MESS discharge power
${{\text{P}}}_{{\text{MESS}}-{\text{charge}}}^{{\text{max}}}$: MESS maximum charging power
${{\text{P}}}_{{\text{MESS}}-{\text{discharge}}}^{{\text{max}}}$: MESS maximum discharging power
${{\text{P}}}_{{\text{MS}}}$: Mobile storage power
${{\text{P}}}_{{\text{MT}}}$: Micro-tuebine power
PV: Photovoltaic
PPV,rated: Photovoltaic rated power
PWT: WT power
RC: Residential consumer
${{\text{RC}}}_{{\text{t}}}^{{\text{max}}}$: Reduction of the maximum demand recommended by residential consumer in period t
S: Irradiance
Sref: Reference irradiance
SOC: State of charge of the MESS
SOCmax: Maximum limit of SOC
SOCmin: Minimum limit of SOC
SOE: Available energy of the MESS
vcutin: Cut-in wind speed
vcutout: Cut-out wind speed
vW: Wind speed
WT: Wind turbine
$\overrightarrow{X}$: Vector of the position of the wolf
${\overrightarrow{X}}_{P}$: Position vector of the prey
${\mu }_{U}$: Mean of the output variable
${\mu }_{Z}$: Value of membership function z
$${\mu }_{{z}_{c}}$$: Mean of $${z}_{c}$$
${\upeta }_{{\text{MESS}}}^{{\text{dch}}}$: MESS discharging efficiency
${\upeta }_{{\text{MESS}}}^{{\text{ch}}}$: MESS charging efficiency
${\sigma }_{U}$: Standard deviation of the output variable
${\sigma }_{{z}_{c}}$: Standard deviation of ${z}_{c}$
${\zeta }_{c,j}$: Standard places of the random input variable
${\lambda }_{c,3}$: Kewness of the random input variable ${z}_{c}$
