# Author Correction: Stochastic energy management of a microgrid incorporating two-point estimation method, mobile storage, and fuzzy multi-objective enhanced grey wolf optimizer

**DOI:** 10.1038/s41598-024-53394-5

**Published:** 2024-02-07

**Authors:** Serajuddin Habibi, Reza Effatnejad, Mahdi Hedayati, Payman Hajihosseini

**Affiliations:** Department of Electrical Engineering, Karaj Branch, Islamic Azad University, Karaj, Iran

Correction to: *Scientific Reports* 10.1038/s41598-024-51166-9, published online 18 January 2024

The original version of this Article contained an error in the affiliation, which was incorrectly given as ‘Department of Electrical Engineering, Islamic Azad University, Karaj Branch, Karaj, Iran’. The correct affiliation is listed below:

Department of Electrical Engineering, Karaj Branch, Islamic Azad University, Karaj, Iran.

The original Article has been corrected.

