# Supplementary material for: Stochastic energy management of a microgrid incorporating two-point estimation method, mobile storage, and fuzzy multi-objective enhanced grey wolf optimizer
Source: Sci Rep. 2024 Jan 18;14:1667. doi: 10.1038/s41598-024-51166-9 (PMC10796331; doi:10.1038/s41598-024-51166-9)
Supplement: Supplementary file 1 — Supplementary Information. [file 41598_2024_51166_MOESM1_ESM.docx]

**Appendix A.** 33-bus microgrid load data

**Table A1.** Load data of 33-bus micro-grid

| Bus Number | Reactive Load, Q (kVAr) | Active Load, P (kW) | Bus Number | Reactive Load, Q (kVAr) | Active Load, P (kW) |
| --- | --- | --- | --- | --- | --- |
| 1 | 0 | 0 | 18 | 18 | 8 |
| 2 | 20 | 12 | 19 | 18 | 8 |
| 3 | 18 | 8 | 20 | 18 | 8 |
| 4 | 24 | 16 | 21 | 18 | 8 |
| 5 | 12 | 6 | 22 | 18 | 8 |
| 6 | 12 | 4 | 23 | 18 | 10 |
| 7 | 40 | 20 | 24 | 84 | 40 |
| 8 | 40 | 20 | 25 | 84 | 40 |
| 9 | 12 | 4 | 26 | 12 | 5 |
| 10 | 12 | 4 | 27 | 12 | 5 |
| 11 | 9 | 6 | 28 | 12 | 4 |
| 12 | 12 | 7 | 29 | 24 | 14 |
| 13 | 12 | 7 | 30 | 40 | 120 |
| 14 | 24 | 16 | 31 | 30 | 14 |
| 15 | 12 | 2 | 32 | 42 | 20 |
| 16 | 12 | 4 | 33 | 12 | 8 |
| 17 | 12 | 4 |  |  |  |
